# Cryo-EM Structure of Recombinantly Expressed hUGDH Unveils a Hidden, Alternative Allosteric Inhibitor

**DOI:** 10.1021/acs.biochem.4c00555

**Published:** 2024-12-16

**Authors:** John H. O’Brien, Renuka Kadirvelraj, Po-Sen Tseng, Nolan Ross-Kemppinen, David Crich, Richard M. Walsh, Zachary A. Wood

**Affiliations:** † Department of Biochemistry & Molecular Biology, University of Georgia, Athens, Georgia 30602, United States; ‡ Department of Pharmaceutical and Biomedical Sciences, Department of Chemistry, and Complex Carbohydrate Research Center, University of Georgia, Athens, Georgia 30602, United States; § Department of Biological Chemistry and Molecular Pharmacology, Blavatnik Institute, Harvard Medical School, Boston, Massachusetts 02115, United States

## Abstract

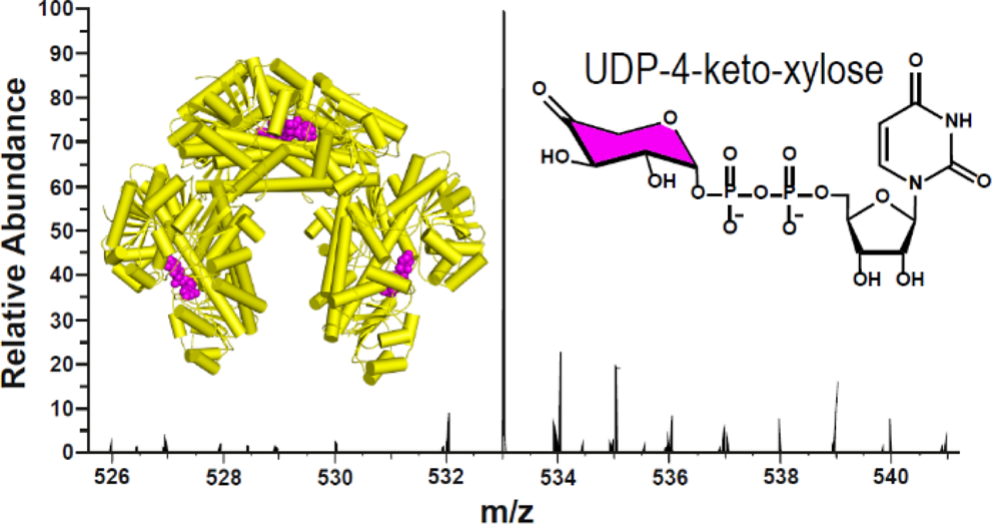

Human UDP-glucose dehydrogenase (hUGDH) catalyzes the oxidation of UDP-glucose into UDP-glucuronic acid, an essential substrate in the Phase II metabolism of drugs. hUGDH is a hexamer that exists in an equilibrium between an active (E) state and an inactive (E^Ω^) state, with the latter being stabilized by the binding of the allosteric inhibitor UDP-xylose (UDP-Xyl). The allosteric transition between E^Ω^ and E is slow and can be observed as a lag in progress curves. Previous analysis of the lag suggested that unliganded hUGDH exists mainly as E^Ω^, but two unique crystal forms suggest that the enzyme favors the E state. Resolving this discrepancy is necessary to fully understand the allosteric mechanism of hUGDH. Here, we used cryo-EM to show that recombinant hUGDH expressed in *Escherichia coli* copurifies with UDP-4-keto-xylose (UX4O), which mimics the UDP-Xyl inhibitor and favors the E^Ω^ state. Cryo-EM studies show that removing UX4O from hUGDH shifts the ensemble to favor the E state. This shift is consistent with progress curve analysis, which shows the absence of a lag for unliganded hUGDH. Inhibition studies show that hUGDH has similar affinities for UDP-Xyl and UX4O. The discovery that UX4O inhibits allosteric hUGDH suggests that UX4O may be the physiologically relevant inhibitor of allosteric UGDHs in bacteria that do not make UDP-Xyl.

## Introduction

UDP-glucuronic acid (UDP-GlcA) is the essential substrate in glucuronidation, a major component of Phase II metabolism of drugs in mammalian cells.^1−3^ Glucuronidation is carried out by glucuronosyltransferases, which catalyze the addition of glucuronic acid to toxins or drugs to be cleared from the body.^4^ Given its role in drug metabolism, it is not surprising that some cancers have co-opted glucuronidation as an effective chemotherapeutic resistance mechanism.^5−8^ Thus, controlling glucuronidation is a promising strategy for treating cancers that exhibit this type of drug resistance. Designing inhibitors to glucuronosyltransferases is a significant challenge because there are 28 human isozymes, which are all integral ER membrane proteins with broad substrate specificity.^4^ An alternative strategy for inhibiting glucuronidation would be to limit the availability of the essential substrate, UDP-GlcA.^9^ In humans, UDP-GlcA is synthesized by UDP-glucose dehydrogenase (hUGDH), which catalyzes the NAD^+^-dependent oxidation of UDP-glucose (UDP-Glc).^10^ The activity of hUGDH is allosterically controlled by UDP-Xyl, a downstream metabolite of UDP-GlcA. Understanding this allosteric mechanism could provide the framework necessary to achieve the important goal of controlling glucuronidation.

**Figure 1:**
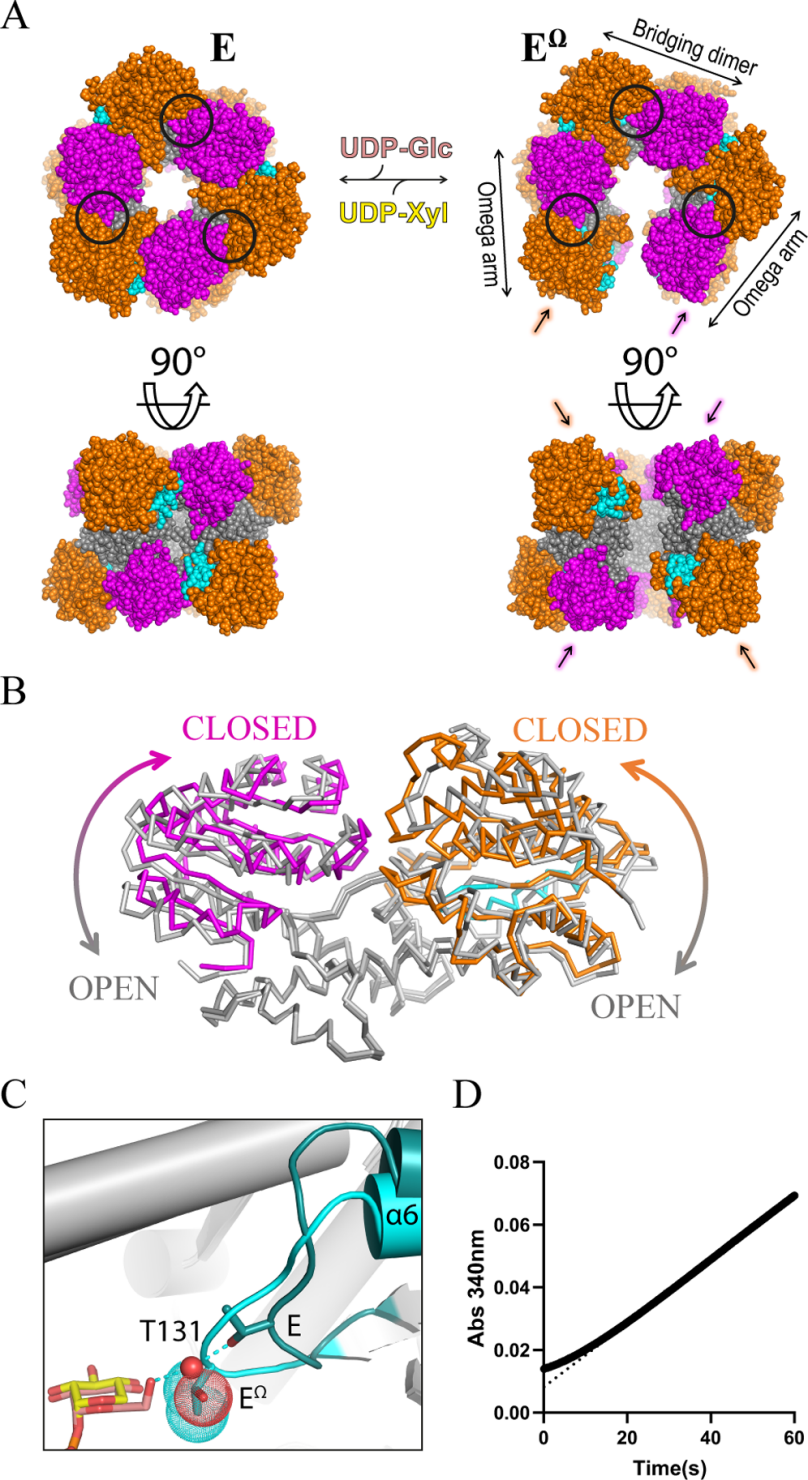
Allosteric regulation of hUGDH. (A) Active E and allosterically inhibited E^Ω^ states of hUGDH (PDB IDs 2Q3E and 5VR8, respectively). In E^Ω^, the dimers with one solvent-exposed domain are called the Omega arms, and the dimers with both domains buried are called the Bridging dimers. The solvent-exposed domains in the broken interfaces are labeled with correspondingly colored arrows. Orange: NAD^+^ binding (NB) domains; Magenta: Substrate Binding (SB) domains; Gray: dimerization domains; Cyan: allosteric switch. The active sites are located between the NB and SB domains of each chain (black circles). (B) Superposition of an open (6C4K, light gray) and closed hUGDH monomer (5VR8, colored as in A). (C) Close up of allosteric switch in the E (teal) and E^Ω^ (cyan) state. UDP-Xyl is shown in yellow, UDP-Glc in salmon. The VDW radii (dots) of Thr131 in E^Ω^ and coordinating water (red sphere) in the E state are shown. (D) Hysteresis in progress curves of inhibited hUGDH. The dotted line highlights the lag in the early time points.

hUGDH forms a hexamer arranged as a trimer of dimers, with each chain containing a NAD^+^ binding domain (NB domain) and a substrate binding domain (SB domain) (Figure 1A).^11^ The active site is located between the NB and SB domains within each chain, which are both flexible and undergo a rigid-body rotation to open the active site and bind substrate (Figure 1B). Binding of the substrate, UDP-Glc, favors the formation of an active, 32 symmetry hexamer called the E state (Figure 1A).The allosteric mechanism of hUGDH is atypical because UDP-Glc and the feedback inhibitor UDP-Xyl compete for the same site, but produce different hexameric complexes. What makes this allosteric is that the binding of UDP-Xyl changes the structure of the hexamer-building interface and increases the affinity between the adjacent subunits to form E^Ω^.^12^ (Figure 1A). This remarkable conformational change is controlled by a buried allosteric switch (the Thr131 loop/α6 helix) that responds to the identity of the C5 position of the nucleotide sugar bound in the active site (Figure 1C).^12^ The C5 hydroxymethyl in UDP-Glc stabilizes the allosteric switch favoring the E state, while UDP-Xyl lacks the C5 hydroxymethyl which induces the repacking of the switch into the E^Ω^ state. The α6 helix of the allosteric switch is buried in the hexamer-building interface, where it controls the structure of the complex and the affinity between adjacent subunits.

The transition from E^Ω^ to E is slow and can be observed as hysteresis in progress curves (Figure 1D).^13−18^ Analysis of the hysteresis in progress curves suggests that unliganded hUGDH favors the E^Ω^ state at pH 7.5, with a E/E^Ω^ ratio of about 1:9.^14^ In contrast, analysis of two different crystal structures suggests that unliganded UGDH favors the E state near physiological pH.^14,17,19^ To resolve the discrepancy between the kinetic and crystallographic studies, we have attempted to approximate the unliganded E:E^Ω^ ratio by solving the cryo-EM structure of hUGDH and quantifying the number of E and E^Ω^ particles. To our surprise, the cryo-EM reconstruction showed that *Escherichia coli*, expressed hUGDH copurifies with a nucleotide sugar which stabilizes the hexamer in the E^Ω^ conformation. Structural and biochemical analyses identify the nucleotide sugar as UDP-4-keto-xylose (UX4O), a structural analogue of UDP-Xyl that is produced in *E. coli*. Our results show that UX4O and UDP-Xyl have similar affinities for hUGDH and both stabilize the inactive E^Ω^ state. We have developed a protocol for producing unliganded hUGDH from *E. coli* and using cryo-EM we show that hUGDH strongly favors the E state in the absence of UX4O.

## Materials and Methods

### Purification of the hUGDH:UX4O Complex and Unliganded hUGDH

Recombinant hUGDH (UniProtKB entry O60701) with an N-terminal histidine tag was expressed in *E. coli* and purified over a TALON column as previously described,^12^ with the following modifications. To purify the hUGDH:UX4O complex, the protein was washed to baseline as usual, using 300 mM NaCl, 5 mM imidazole, 50 mM Na/K phosphate (pH 7.8), followed by 1.0 M NaCl, 50 mM Na/K phosphate (pH 7.8), and finally with 10% glycerol, 50 mM Na/K phosphate (pH 7.8). To purify unliganded hUGDH, we included a final wash step of 50 mM sodium tetraborate (pH 9.2), 100 mM NaCl to elute the UX4O. The UX4O elution peak was collected and saved for additional purification and analysis. The washed hUGDH:UX4O or hUGDH was then eluted with 300 mM imidazole, 50 mM Na/K phosphate (pH 7.8), dialyzed into 300 mM NaCl, 1 mM TCEP, 15 mM Tris (pH 8.0), and digested with tobacco etch virus^20^ protease to remove the histidine tag. Finally, both hUGDH:UX4O and hUGDH were dialyzed into 50 mM NaCl, 10 mM Tris (pH 8.0) and concentrated to 20 mg/mL. Protein purified with or without the sodium tetraborate wash have equivalent specific activities, indicating that the high pH borate wash did not denature the protein (discussed in Results). The eluted UX4O was purified and analyzed as described below.

### Cryo-EM Grid Preparation, Data Collection, Image Processing, and Structure Refinement

In preparation for cryo-EM work, the hUGDH:UX4O and hUGDH samples were polished using a Superdex 200 column (Biorad) pre-equilibrated with 20 mM Hepes pH 7.5, 100 mM NaCl and 1 mM DTT. The hUGDH:UX4O sample was directly deposited on cryo-EM grids at a concentration of 1 mg/mL, but the unliganded hUGDH was supplemented with Fos-choline-8, fluorinated solution (Anatrace) to a final concentration of 0.7 mM and 2.2 mg/mL protein prior to disposition. All samples were deposited on 300 mesh Quantifoil Au 0.6/1.0 grids that were glow discharged in a PELCO easiGLOW (Ted Pella) at 0.39 mbar, 15 mA for 30 s and vitrified in liquid ethane using a Vitrobot Mark IV (Thermo Fisher Scientific), with a wait time of 8 s, blot time of 7–8 s and a blot force of six at 22 °C at 100% humidity.

Micrographs were recorded on a 300 kV Titan Krios G3i microscope with either a K3/Bioquantum (Gatan) or Falcon4i/Selectris (Thermo Fisher Scientific) for the nucleotide-bound and unliganded sample, respectively. Data were collected using SerialEM^21^ or EPU (Thermo Fisher Scientific) automated collection software packages for the nucleotide-bound and unliganded sample, respectively. Dose-fractionated images were gain normalized, aligned, dose-weighted, and summed using MotionCor2^22^ Contrast transfer function (CTF) and defocus value estimation were performed using CTFFIND4.^23^ Details of the data processing schemes are summarized in the Supplementary Data (SI Figures 1–4) and in Table 1. Structural biology applications for image processing used in this project were compiled and configured by SBGrid.^24^

**Table 1:** Cryo-EM Data Collection, Refinement, and Validation Statistics

Cryo-EM data collection and processing	hUGDH:UX4O	hUGDH
PDB code	9DH0	9DGZ
EMDB code	EMD-46854	EMD-46853
nominal magnification	105 kX	165 kX
voltage (kV)	300	300
electron exposure (e^–^/Å^2^)	54.42	46.44
defocus range (μm)	0.8, 2.2	0.8, 2.2
pixel size (Å)	0.825	0.73
symmetry imposed	C2	D3
initial particle images (no.)	4,118,676	1,786,206
final particle images (no.)	3,059,917	918,122
map resolution (Å)	2.38	2.06
map resolution range (Å)	2.12–4.756	2.0–2.5
FSC threshold	0.143	0.143
**refinement**		
initial model used	5VR8	5TJH
model resolution (Å)	2.5	2.1
FSC threshold	0.5	0.5
map-sharpening *B*-factor (Å^2^)	–106.8	–62.73
number of atoms: protein/ligand/water	18194/132/193	21556/-/470
mean *B*-factor (Å^2^): protein/ligand/water	114.34/105/106	82.4/-/84.4
R.M.S deviations: bond lengths (Å)/bond angles (deg)	0.005/1.1	0.003/0.49
**validation**		
molprobity score	1.2	1.12
clash score	4.1	3.3
rotamer outliers (%)	0.6	0.51
Ramachandran plot		
favored (%)	97.95	98.27
allowed (%)	2.05	1.73
disallowed (%)	0.00	0.00

For the nucleotide-bound sample, particle picking was carried out from 13,500 micrographs in crYOLO,^25^ generating 4,118,676 particles in the initial data set. This was followed by heterogeneous refinement in CryoSPARC^26^ generating a single class with 3,059,917 particles. These data were then subjected to iterative 3D and CTF refinements followed by Bayesian polishing in Relion. Polished particles were then subjected to Nonuniform refinement and local refinement in CryoSPARC. These data generated a C2 reconstruction at 2.38 Å where the NAD domains for the two arm dimers were disordered (SI Figures 1 and 2). To obtain better order for all NAD domains, we used focused 3D classification without alignment in C1 to identify a subset of 166,809 particles (∼5% of the full data set). This data was also subjected to Nonuniform refinement and Local refinement generating a final reconstruction at 2.93 Å with C1 symmetry (SI Figures 1 and 2) with ordered NAD domains for all dimers. However, due to the lower resolution of the map, we concentrated our analysis on the higher resolution model (2.38 Å) from the full data set.

For the unliganded sample, particle picking was carried out from 35,333 micrographs in Topaz^27^ generating 1,786,206 particles in the initial data set. Iterative 2D classification identified 918,122 good particles that were used in the final data set (SI Figure 3). These data were then subjected to Bayesian polishing, iterative CTF refinement and 3D refinement with Blush regularization^28^ producing a 2.06 Å reconstruction with D3 symmetry (SI Figure 4). All data processing for the unliganded data set were carried out in Relion.^28,29^

To refine the unliganded hUGDH cryo-EM model, the crystal structure of the hUGDH_A136M_ hexamer (PDB ID 5TJH) was manually fit into the cryo-EM density map using ChimeraX^30^ followed by rigid-body fitting of each monomer into the map using Coot.^31^ Refinement was carried out using Phenix^32^ where the NAD^+^ and UDP-Glc binding domains of the hUGDH hexamer were first subjected to rigid-body refinement. This was followed by subsequent cycles of real-space refinement with macrocycles including morphing (as implemented in Phenix), global minimization, ADP, Ramachandran and reference model restraints. These strategies combined with model adjustments using Coot^31^ led to the final model (Table 1). To refine the nucleotide-bound hUGDH structure, the crystal structure of UDP-Xyl bound hUGDH hexamer (PDB ID 5VR8) was manually fit into the cryo-EM density map using ChimeraX^30^ and then refined in the same manner as the unliganded protein (Table 1).

### Determining the UDP-4-Keto-xylose (UX4O) Binding Stoichiometry

The binding stoichiometry of the UDP-4-keto-xylose that copurified with hUGDH was determined using a previously described protocol.^18^ First, the absorbance spectrum of recombinantly expressed hUGDH (6.8 mg/mL) was recorded on an Agilent 8453 UV/vis Spectrometer. Next, the sample was recovered from the cuvette, boiled for 1 min, then cooled and centrifuged to remove the precipitated protein. The UV-spectra of the clear supernatant revealed an absorbance peak at 262 nm, consistent with a nucleotide sugar, and was quantified using the molar absorptivity of uridine (ε_262_ = 9820 M^–1^ cm^–1^) (Figure 3C). To quantify the amount of protein present in the sample, the spectrum of the nucleotide sugar was subtracted from that of the initial UX4O-bound hUGDH. The resulting spectrum represents the unliganded hUGDH, which was quantified using the molar absorptivity of ε_280_ = 50,600 M^–1^ cm^–1^ calculated from ProtParam.^33^ The stoichiometry of binding was then calculated by dividing the molar concentration of the UX4O by the concentration of hUGDH.

### ArnA Synthesis of UX4O

The decarboxylase domain of ArnA was used to synthesize UX4O, to use as both a standard and a reagent in inhibition studies.^18,34,35^ Briefly, 40 μM of the ArnA decarboxylase domain in reaction buffer (25 mM Tris pH 8.0 and 50 mM NaCl) was added to a 2.9 mL solution containing 12 mM UDP-GlcA and 8 mM NAD^+^. We have previously shown^18^ that ArnA will produce small amounts of UDP-Xyl in the reaction when UDP-GlcA is depleted and the UX4O and NADH products rebind to the enzyme, but this secondary reaction is inefficient and can be avoided by using a molar excess of UDP-GlcA over NAD^+^. The ArnA reaction was allowed to proceed until the NADH production plateaued, as determined by the change in absorbance at 340 nm. The reaction was passed through a 10 kDa cutoff spin filter to remove the enzyme, and the reaction products were resolved using an ÄKTA Pure FPLC system equipped with a 5 mL Cytiva HiTrap Q HP anion exchange column. UX4O was purified using gradient elution of 20 mM ammonium acetate, pH 7.0 (buffer A) and 0.5 M ammonium acetate pH 7.0 (buffer B). UX4O was eluted with 40% buffer B, concentrated to 0.5 mL, and desalted with G10-120 size exclusion resin as described below. The purified sample was concentrated to 0.5 mM and stored at −80 °C. We found that UX4O is unstable when dried to completion, so it was always kept in aqueous solution during purification and analysis.

### Purification and HPLC Analysis of UX4O

The UX4O that was eluted from hUGDH was desalted using an Extract-Clean Carbograph SPE column (150 mg Graphitized Carbon). The Carbo column was first equilibrated with 3 mL of room temperature 80% acetonitrile containing 0.1% trifluoroacetic acid, followed by 2 mL of nano pure water. The borate fraction from hUGDH was adjusted to pH 4.8 using glacial acetic acid and then added to the prepared Carbo column. The column was then washed with 0.5 mL of 10 mM ammonium acetate pH 4.8 to remove the borate, and the nucleotide was step-eluted using 0.25 mL fractions of a solution of 65% acetonitrile containing 50 mM triethylammonium acetate as the ion pairing agent. The four fractions with the highest concentrations of nucleotide sugar were identified based on absorbance at 262 nm (Agilent 8453 UV–vis spectrometer) and pooled. The pooled sample (1 mL) was then loaded into an ÄKTA Start FPLC through a 1 mL sample loop. The FPLC was equipped with an Amersham Biosciences adjustable XK16 column, packed with Sephadex G10-120 size exclusion resin of dimensions 16 mm × 138 mm, and equilibrated with deionized water. Deionized water was also used as the running buffer. The FPLC was operated at a flow velocity of 15 cm/h, corresponding to a volumetric flow rate of 0.5 mL/min. The fractions corresponding to a 260 nm absorbance signal were collected in 0.5 mL increments and pooled. The sample was concentrated to 0.5 mM using an Eppendorf Speedvac and stored at −80 °C. It is important to avoid drying the UX40 to prevent it from reacting with nucleophiles. As described in the results, the UX4O exists as a stable geminal-diol in water (UDP-4-geminal-diol-xylose), but upon drying, the C4 position will dehydrate and the 4-keto group is reactive.

Purified UX4O was analyzed using an Agilent 1260 Infinity II HPLC system with a Dionex Carbopac PA20 column (3 mm × 150 mm) equipped with a Dionex AminoTrap Guard column (3 mm × 30 mm). UDP-Xylose (Carbosource, CCRC at the University of Georgia) was added as a control. The column was equilibrated with 50 mM ammonium formate (pH 5.0) and the samples were eluted using a linear gradient of 1 M ammonium formate (pH 5.0) using a flow rate of 0.5 mL/min at room temperature. The nucleotide signals were monitored at 262 nm. UDP-Xylose and UX4O were easily resolved, eluting at 11.4 and 11.7 min, respectively.

### NMR and Mass Spectrometry Identification of UX4O

The putative UX4O sample (1 mg) and a known UX4O standard (2 mg) were each dissolved to a total of 0.6 mL using 90% D_2_O in 5 mm NMR tubes. ^1^H NMR spectra were recorded at 600 MHz at 25 °C on a Bruker AVANCE NEO 600 MHz spectrometer equipped with a 5 mm TCI cryoprobe, and chemical shifts are referenced to residual HOD at 4.79 ppm. ^13^C NMR spectra were ^1^H decoupled and were recorded at 151 MHz at 25 °C on a Bruker AVANCE III 600 MHz spectrometer equipped with a 5 mm TCI cryoprobe. Peak assignments were based on two-dimensional NMR (^1^H–^1^H COSY, ^1^H–^13^C HSQC and ^1^H–^13^C HMBC) experiments, which were performed at 25 °C on a Bruker AVANCE III 600 MHz spectrometer equipped with a 5 mm TCI cryoprobe. Data were analyzed using MestReNova v14.2.3. All NMR experiments are summarized in SI Figure 5.

High-resolution electrospray ionization mass spectrometry spectra were recorded using a Thermo Scientific Orbitrap mass analyzer. Data were analyzed using the Thermo Scientific FreeStyle 1.7 software.

### Sedimentation Velocity

Sedimentation velocity experiments were conducted, as previously described.^12,14−17,36^ Briefly, hUGDH was dialyzed for 12 h at 21 °C into a buffer containing 25 mM Hepes pH 7.5 and 150 mM KCl. The enzyme concentration was set to 4.5 μM with the same buffer after dialysis. Enzyme samples were loaded into analytical ultracentrifugation cells equipped with 12 mm double-sector Epon centerpieces and quartz windows. Cells were torqued and loaded into an An60 Ti rotor to first be equilibrated to 20 °C for 1.5 h. Sedimentation velocity data were collected using an Optima XLA analytical ultracentrifuge at 50,000 rpm for 8–10 h. Absorbance data were recorded at 280 nm in radial step sizes of 0.003 cm. SEDENTERP was used to estimate the partial specific volume of hUGDH (0.740497 mL g^–1^), the buffer density (1.0079 g mL^–1^), and viscosity (0.0101838 P).^37^ Data were modeled as a continuous sedimentation coefficient (c(s)) distribution using SEDFIT.^38^ The baseline, meniscus, frictional coefficient, systematic time-invariant noise, and radial invariant noise were fit.^39^

### Enzyme Kinetics

All steady-state kinetic assays were performed as previously described.^13−17,19,40^ Briefly, 100 nM of enzyme was used in a standard reaction buffer containing 50 mM Hepes pH 7.5, 50 mM NaCl, 5 mM EDTA, and 0.01% Tween-20 with either saturating levels of NAD^+^ (5 mM, Sigma) or UDP-Glc (1 mM, Sigma). Substrate and enzyme were preincubated separately at 25 °C for 5 min before starting the reaction. For the Substrate Saturation curves (SSCs), samples were rapidly mixed by hand and run on an Agilent 8453 UV/vis Spectrometer equipped with a Peltier temperature controller (25 °C). NADH production was continuously monitored at 340 nm (molar absorptivity coefficient of 6,220 M^–1^ cm^–1^). Progress curves of hUGDH display hysteresis, thus the observed initial velocity (*v*_*i*_) represents a transient and does not satisfy steady-state conditions. To obtain steady-state initial velocities (*v*_*ss*_), progress curves before the depletion of 10% of the substrate were fit to Frieden’s equation^41^ as in previous studies:^13−17,19,40^
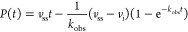
1Where *P* is the product produced at time *t*, and *k*_obs_ is the apparent rate constant for the transition between the initial (*v*_*i*_) and final steady-state velocities (*v*_ss_). The *v*_ss_ was used for determination of hUGDH steady-state kinetic parameters.

Data were fit using nonlinear regression analysis in PRISM (GraphPad Software). To report *k*_cat_ values, *V*_ss_ was normalized to *V*_ss_/[*E*]. The resulting SSCs were fit to either a hyperbolic or sigmoidal model^42^ using eq 2 based on residual analysis.
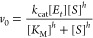
2For inhibition studies, UDP-Xyl or UX4O were preincubated with enzyme for 5 min at 25 °C prior to initiating the reaction. The *K*_*i*_ for each inhibitor was determined using global fitting of at least three substrate saturation curves with different concentrations of inhibitor to eq 2. *K*_M_ value in eq 2 was replaced with eq 3 prior to global fitting.
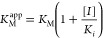
3The negative cooperativity observed in hUGDH NAD^+^ saturation curves was analyzed using Kurganov’s equation as described previously.^15,16,43^ The data were fit to eq 4.
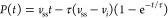
4where *K*_0_ is the approximate *K*_M_ for the high-affinity binding sites and *K*_lim_ is the average apparent *K*_M_ for the low affinity sites.^43^

Hysteresis in progress curves was analyzed with an Applied Photophysics SX20 stopped-flow spectrophotometer. For the hUGDH and hUGDH:UX4O experiments, 100 nM of enzyme was rapidly mixed with saturating amounts of substrate (5 mM NAD^+^ and 400 μM UDP-Glc) at 25 °C. The NADH signal was continuously monitored at 340 nm, as above. To quantify the impact of UDP-Xyl on the lag of purified hUGDH, the enzyme was preincubated with 1 μM of exogenous UDP-Xyl for 5 min at 25 °C before starting the reaction.

## Results

### *E. coli*-Expressed hUGDH Favors the E^Ω^ Conformation

**Figure 2:**
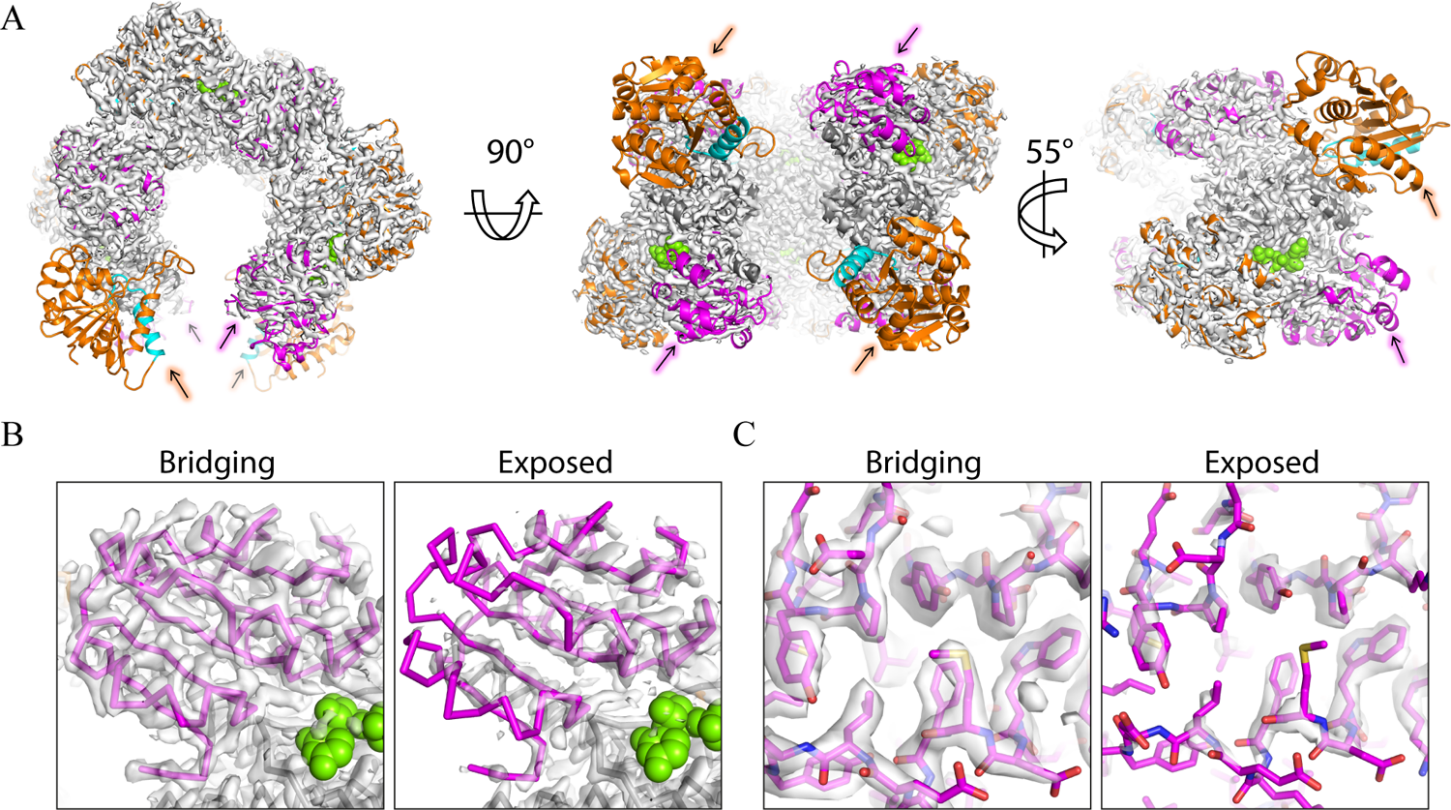
Cryo-EM model of hexameric hUGDH:UX4O shows flexibility in the NB_exposed_ and SB_exposed_ domains. (A) Cryo-EM density map of hUGDH:UX4O (5σ, light gray) with the cartoon model colored by domain. The bound UX4O that copurified with the enzyme is shown in green (see text). The NB Domains (orange), SB domains (magenta), dimerization domains (dark gray), and allosteric switch (cyan) are shown. The correspondingly colored arrows identify the flexible, solvent-exposed NB and SB domains located in the Omega arms of the complex. The NB_exposed_ domains were not observed in the initial reconstruction and are modeled from the superimposed E^Ω^ crystal structure, PDB ID 5VR8. (B) A well-ordered SB domain from the bridging dimer and a flexible S*B*_exposed_ domain from the Omega arm dimer. (C) Representative densities of B.

hUGDH was expressed in *E. coli* and electron microscopy grids were prepared. Single particle cryo-EM data was collected and processed, revealing one major class corresponding to the E^Ω^ conformation with a global resolution of 2.38 Å (Figures 2A, S1, S2, Table 1). This cryo-EM structure is similar to the previously published UDP-Xyl bound E^Ω^ crystal structures.^12,17^ The most significant differences are observed in the solvent-exposed NB and SB domains (NB_exposed_ and SB_exposed_) in the Omega arms of the E^Ω^ hexamer (Figure 2). The NB domains of hUGDH are known to be flexible and can rotate between an “open” conformation for binding NAD^+^ and a “closed” conformation during turnover (Figure 1A).^13,14,36,40^ The lack of density for the NB_exposed_ domains and weak density for the SB_exposed_ domains in the cryo-EM structure of the E^Ω^ state shows that they most likely exist as an ensemble of states in solution (Figure 2). In contrast, all crystal structures of the E^Ω^ state show that the NB and SB domains are ordered and in the closed conformation.^12,17^ This discrepancy suggests that the apparent ordering of the NB_exposed_ and SB_exposed_ domains in the earlier crystal structures was a result of crystal packing. In fact, the flexibility of the SB_exposed_ domains has not been observed in the hUGDH crystal structures, though it was predicted in earlier work,^36^ and has been observed in the crystal structures of UGDH from *Caenorhabditis elegans* (61% sequence identity).^19^

### *E. coli*-Expressed hUGDH Copurifies with UDP-4-Keto-xylose

**Figure 3:**
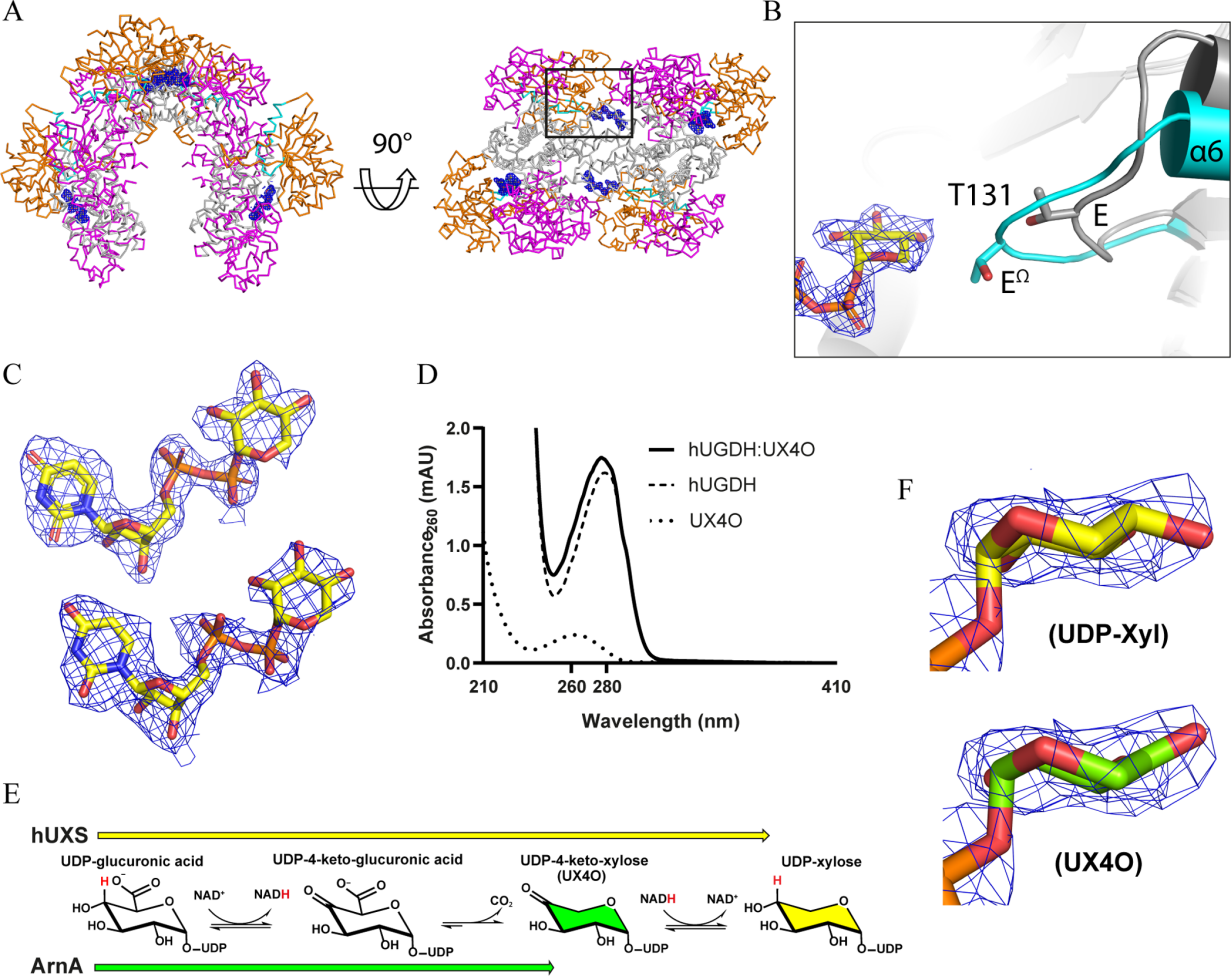
Evidence of UDP-4-keto-Xylose (UX4O) in the hUGDH active site. (A) Cryo-EM structure of the hUGDH:UX4O E^Ω^ complex with density (blue mesh, 4 σ) for the four well-ordered nucleotide sugars in the NB domains. The weak density for the 2 nucleotide sugars in the N*B*_exposed_ domains is not shown. The black square is the area focused on in B. Domain colors same as Figure 1. (B) The allosteric switch in the cryo-EM structure in the E^Ω^ state (cyan) is superimposed on an E state switch from PDB ID 2Q3E (gray). UDP-Xyl was initially modeled in the density. (C) UDP-Xyl modeled in *top*, well-ordered density (5.5 σ) from bridging monomers with ordered NB domains, and *bottom*, weaker density (2 σ) from Omega arm monomers with flexible NB_exposed_ domains. (D) Absorbance spectra of hUGDH:UX4O complex (solid line). Boiling the hUGDH:UX4O sample and clearing the supernatant of protein releases UX4O (dotted line). The unliganded hUGDH spectrum (dashed line) was calculated as the difference between the total protein and UX4O; the molar concentrations were calculated using the appropriate molar absorptivity constants (see Methods). (E) Both ArnA (green arrow) and hUXS (yellow arrow) catalyze the NAD^+^ dependent oxidation of UDP-GlcA to UX4O (green sugar). hUXS reduces UX4O to UDP-Xyl (yellow sugar) while ArnA releases UX4O as product. (F) UDP-Xyl and UX4O modeled in density (5.5 σ).

The active sites of the cryo-EM hUGDH E^Ω^ reconstruction reveal density for six nucleotide sugars that resemble the allosteric inhibitor UDP-Xyl. Similar to UDP-Xyl, these sugars stabilize the allosteric switch in the inhibited state which explains why we observe the E^Ω^ hexamer (Figure 3A–C). Two of the nucleotide sugars are located in the disordered NB_exposed_ domains and have correspondingly weaker density (Figure 3C). To better understand the relationship between the weak nucleotide sugar binding and domain flexibility, we used focused 3D classification to identify a subset of particles (∼5% of the full data set) with closed NB_exposed_ domains (Figures S1 and S2). Refinement generated a final reconstruction at a lower resolution of 2.93 Å. This new reconstruction shows density for the nucleotide sugars in the closed NB_exposed_ domains is comparable to that in the bridging NB domains (not shown). These observations suggest that the increased domain flexibility weakens the binding affinity for the nucleotide sugar.

Because ligands were not added to the protein prior to preparing the electron microscopy grids, the nucleotide sugar likely copurified with the recombinantly expressed enzyme. Thus, we examined a sample of purified protein for the presence of a nucleotide sugar by using a previously described assay^18^ in which the protein is denatured and the amount of nucleotide sugar released into the solvent is measured (see Methods). Assuming the nucleotide sugar is a UDP-Xyl analog, the assay shows that the protein contained the nucleotide sugar with a binding stoichiometry of 0.75, or 4.5 molecules per hexamer (Figure 3D). This is consistent with our observation of four well-ordered and two partially occupied nucleotide sugars based on the cryo-EM structures. Analysis of different protein preps yielded similar results (not shown), suggesting that hUGDH expressed in *E. coli* is prone to copurifying with the contaminating nucleotide sugar.

**Figure 4:**
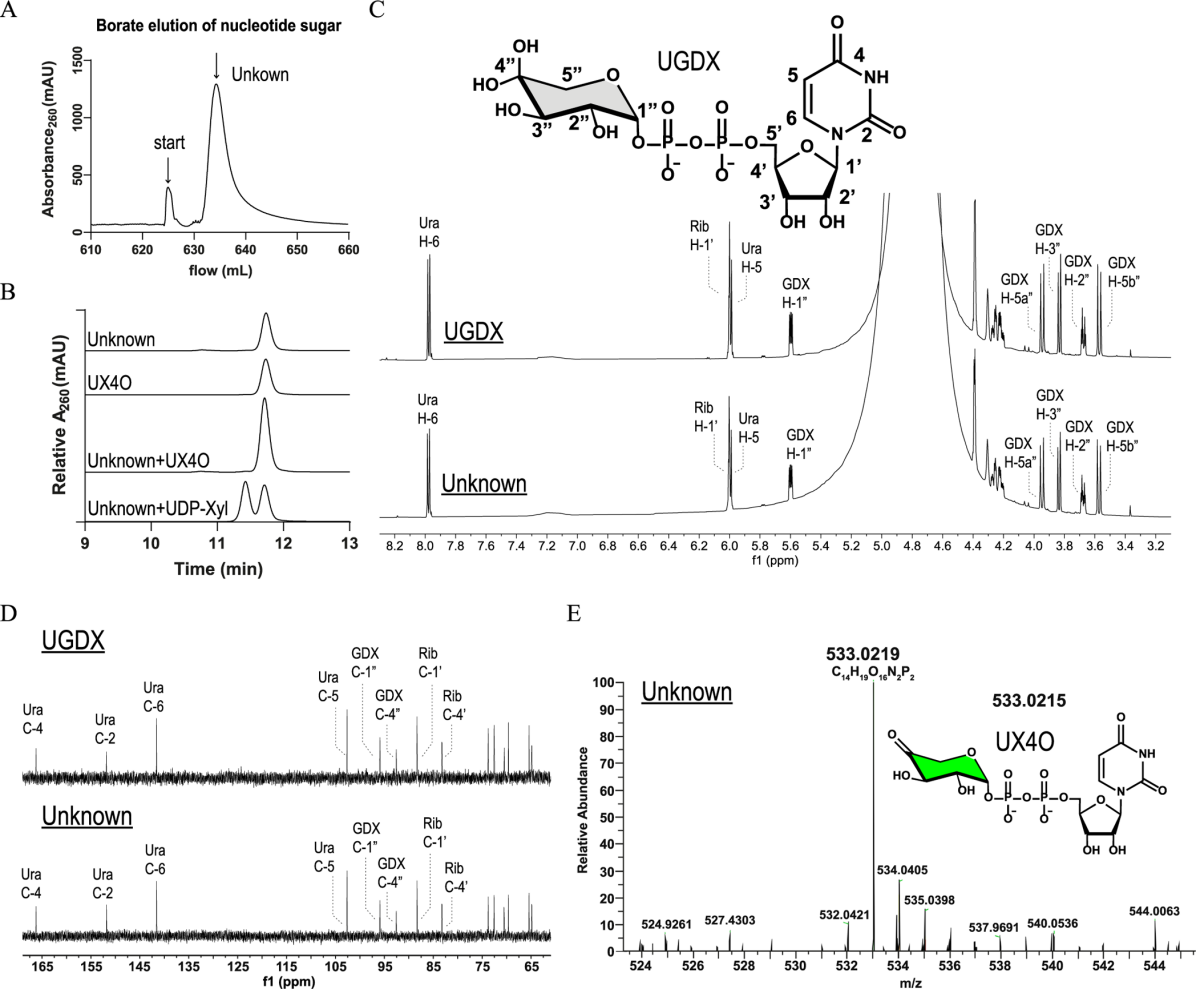
Identification of the nucleotide sugar as UX4O. **(**A) FPLC chromatogram (260 nm) showing the nucleotide sugar (Unknown) eluting from hUGDH with 50 mM sodium tetraborate buffer (pH 9.2). Peak labeled “Start” shows a pressure artifact of stopping and starting the flow of the sodium tetraborate buffer. (B) HPLC chromatograms showing that the purified nucleotide sugar (Unknown) comigrates with the UX4O standard (UX4O) and is distinct from UDP-Xyl (Unknown + UDP-Xyl). (C) Partial ^1^H NMR spectra of the hydrated form of the UX4O standard (UDP-4-geminal-diol-xylose, UGDX) and purified nucleotide sugar (Unknown). The labeled structure of UGDX (inset) (D) Partial ^13^C NMR spectra of UGDX and the purified nucleotide sugar (Unknown). (E) High-resolution electrospray ionization mass spectrum shows evidence of UX4O.

Despite the structural similarity to UDP-Xyl, *E. coli* does not contain UDP-xylose synthase (UXS). However, it does contain a homologous enzyme, ArnA.^18,34,35^ While both enzymes catalyze the first step of the oxidative decarboxylation of UDP-GlcA to produce UDP-4-keto-xylose (UX4O), ArnA releases UX4O as a product whereas UXS reduces the UX4O to produce UDP-Xyl (Figure 3E). Because UX4O and UDP-Xyl are structurally similar, they are both reasonable fits to the density (Figure 3F). To confirm that the nucleotide sugar is UX4O, we purified it from recombinantly expressed hUGDH by taking advantage of the observation that high pH weakens the affinity of hUGDH for UDP-Xyl (see Methods).^14,44^ Briefly, the recombinantly expressed hUGDH was bound to a TALON column and washed with 50 mM sodium borate (pH 9.2) to elute the nucleotide sugar (Figure 4A, see Methods). HPLC analysis reveals that the purified nucleotide sugar comigrates with a known UX4O standard and is easily resolved from a UDP-Xyl standard (Figure 4B). Both the ^1^H and ^13^C NMR spectra of the purified nucleotide sugar also matches the corresponding spectra of aUX4O standard (Figure 4C,D). Analysis of these data and the two-dimensional NMR spectra (Figure S5) reveals that the resonance for pyranose C-4″ appeared at 92.6 ppm in the ^13^C NMR spectra, which indicates that the UX4O samples exist predominantly as the hydrate, UDP-4-geminal-diol-xylose (UGDX). The observation that UX4O favors the gem-diol in aqueous solution has been previously reported by other groups.^34,35,45,46^ High-resolution electrospray ionization mass spectrometry spectrum shows that the gem diol dehydrates to generate the 4-keto-xylose under vacuum (Figure 4E), suggesting that the keto-sugar is the major form when there is no water present.

**Figure 5:**
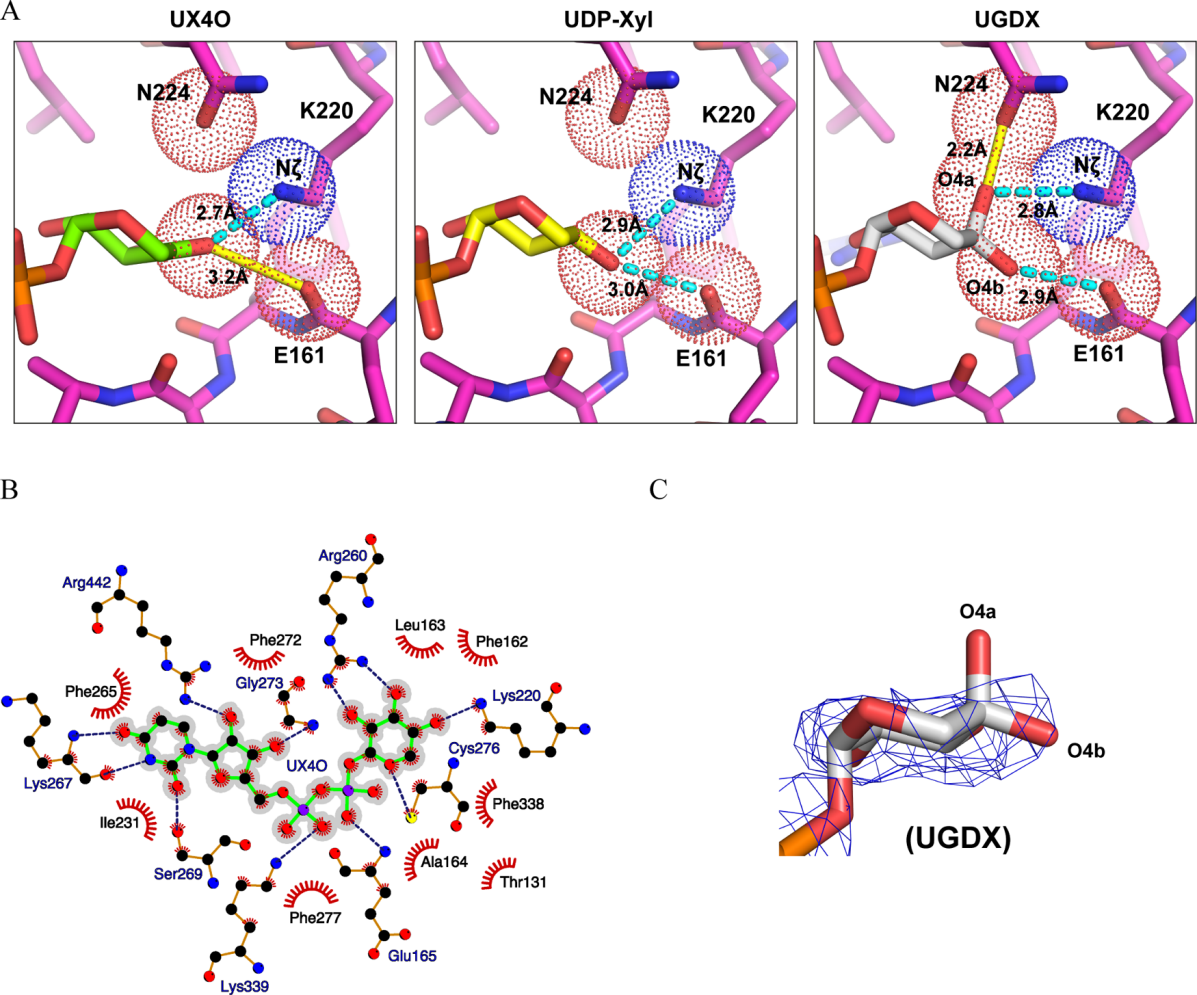
hUGDH active site binds UX4O, but not UGDX. **(**A) The active site with modeled UX4O, UDP-Xyl, and UGDX. VDW radii (dots) and labels are shown for relevant atoms. Dashed cyan lines represent hydrogen bonds. Solid yellow lines represent unfavorable interactions (see text). (B) Ligplot of UX4O. Dashed and feathered lines identify H-bonds and VDW interactions, respectively (C) UGDX modeled into 5.5 σ cryo-EM density.

To identify which form is bound, we attempted to model each in the active site. Modeling UX4O in the active site shows that the 4-keto-sugar fits the density and conserves most of the hydrogen bonding and packing interactions as previously observed for UDP-Xyl^12^ (Figures 5A,B and 3F). The most significant difference between the UDP-Xyl and UX4O interactions involves the backbone carbonyl of E161. The C4 hydroxyl of UDP-Xyl accepts a hydrogen bond from K220 while donating to the E161 carbonyl (Figure 5A); in contrast, the C4 ketone oxygen of UX4O would be expected to experience an unfavorable electrostatic repulsion with the E161 carbonyl oxygen. We attempted to model the gem-diol in the active site, but the best fit of UGDX places the C4 axial hydroxyl (O4a) of the gem diol outside of the density (Figure 5C). In this position, the O4a of UGDX forms a hydrogen bond with K220, but it also introduces a steric clash (2.2 Å) with the carboxamide oxygen of N224 (Figure 5A). N224 is well-ordered, and there is no room for the carboxamide to move and accommodate the UGDX. UGDX is also well packed, and there is no room for it to shift to alleviate the steric clash. Based on the poor fit of UGDX to the density and the unfavorable steric clash, it is likely that the gem diol dehydrates in the active site and that UX4O is the bound form of the nucleotide. Thus, it is possible that the favorable dehydration of the gem-diol in the absence of bulk water may offset the unfavorable electrostatic interaction between the ketone and the E161 carbonyl oxygens observed in the UX4O model (Figure 5A).

### Unliganded hUGDH Favors the E State

**Figure 6:**
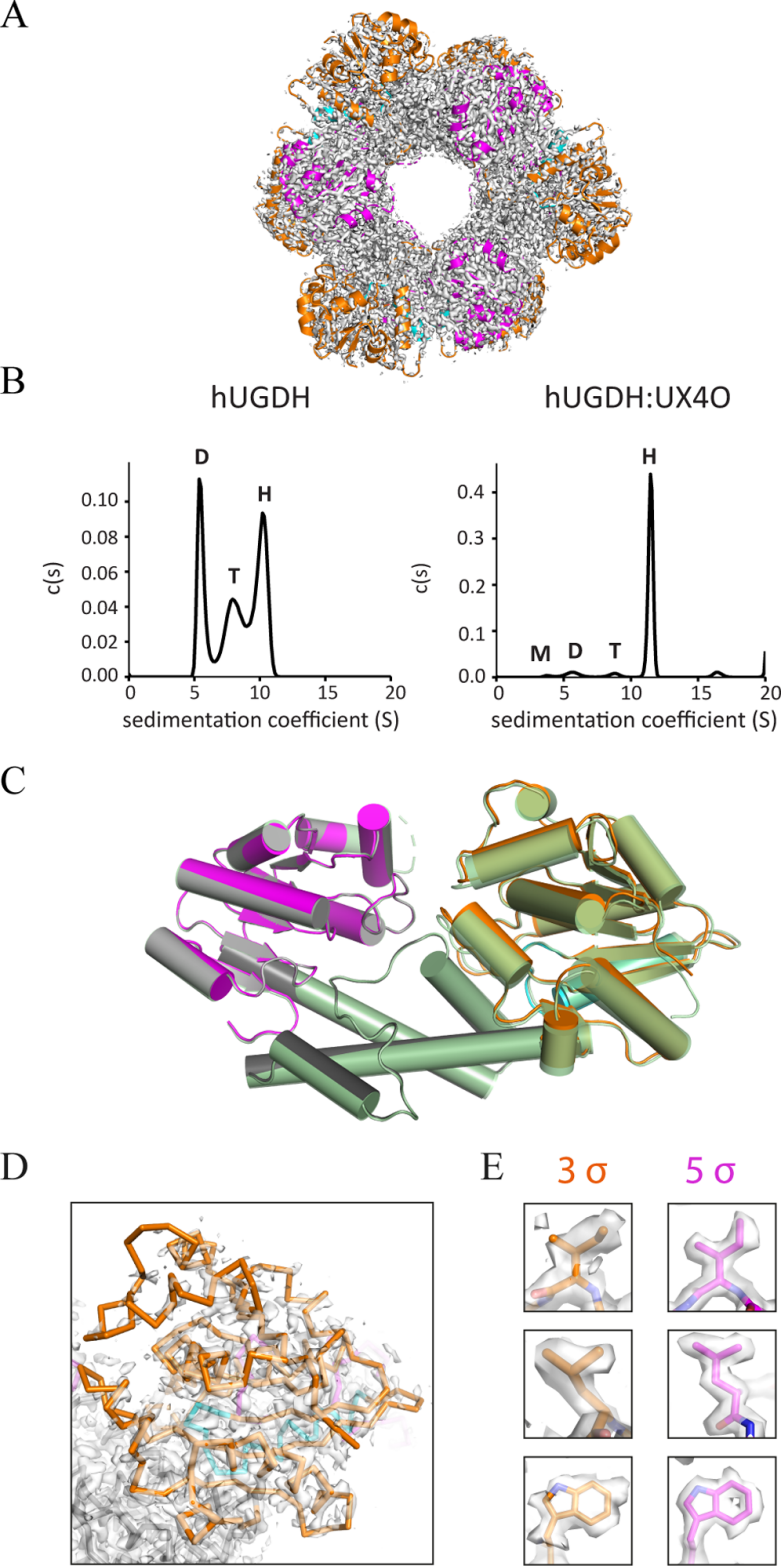
E-state of unliganded hUGDH. (A) Cryo-EM density and structure of hUGDH in the E state identifying the NB (orange) and SB (magenta) domains. (B) Sedimentation velocity data showing that the hUGDH hexamer is less stable than the hUGDH:UX4O complex. (C) hUGDH The cryo-EM model is superimposed on an open domain crystal structure (3ITK, pale green). (D) The cryo-EM density for the NB domains of hUGDH is weakly ordered, indicating domain flexibility. (E) Representative residues from hUGDH with cryo-EM density contoured at the indicated sigma levels. Flexible NB domain (orange), and well-ordered SB domain (purple) are shown.

Unliganded hUGDH (hUGDH) was purified and used to prepare electron microscopy grids for single particle cryo-EM data collection. Processing, refinement, and image reconstruction produced a 2.06 Å density map which revealed that the major class of unliganded hUGDH is the 32 symmetry E state (Figure 6A). Analysis of the particle set with 2D classification shows that the E state represents ∼60% of the particles, with the remaining fraction appearing to represent dimers or monomers, which is consistent with sedimentation velocity data showing that the E state forms a less stable hexamer than the E^Ω^ state (Figure 6B). As expected, there is no evidence of a nucleotide sugar in the active sites (not shown). The cryo-EM structure also shows that all the NB domains are rotated 11.8° to the open conformation (Figure 6C). The cryo-EM density of the open NB domains is less ordered than the rest of the structure, indicating domain flexibility (Figure 6D,E). This is consistent with the various crystal structures of the E state, which reveal NB domain rotations ranging from 0 to 13°, depending on the unique packing constraints of the different crystal lattices.^11,14,17,36^

**Figure 7:**
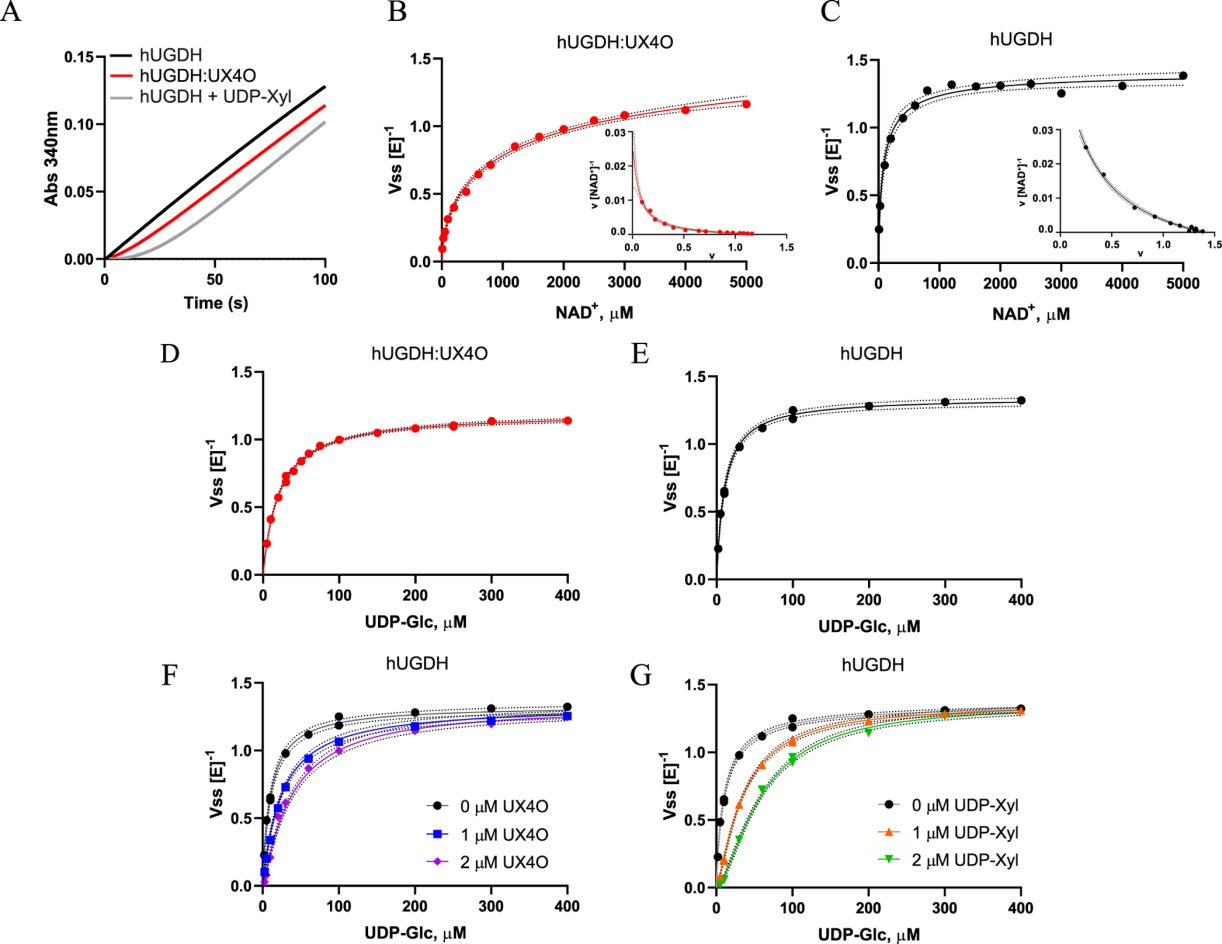
Kinetic characteristics of hUGDH. (A) Progress curves showing the lack of hysteresis in hUGDH (black), the presence of the lag in hUGDH:UX4O (red), and the return of a lag in hUGDH preincubated with 1 μM UDP-Xyl (gray). The differences in the length of the lag are due to differences in saturation (B, C) NAD^+^ substrate saturation curves of hUGDH:UX4O (B) or hUGDH (C) fit to eq 2. Dashed lines represent the 95% confidence interval of the fit and rates are normalized to enzyme concentration. Insets show Eadie-Hofstee plots fit to Kurganov’s eq (eq 4), where concave-up curvature indicates negative cooperativity. (D, E) Hyperbolic UDP-Glc substrate saturation curves with hUGDH:UX4O (D) or hUGDH (E). (F) UDP-Glc substrate saturation curves of hUGDH with 0 μM (black circles), 1 μM (blue squares), and 2 μM (purple diamonds) of UX4O as inhibitor were fit globally to eqs 2 and 3. Dashed lines represent the 95% confidence interval of the fit. (G) Same as in F, but with UDP-Xyl as the inhibitor at 1 μM (orange triangles) and 2 μM (green triangles) concentrations.

Additional evidence that unliganded hUGDH favors the E state can be observed in progress curve analysis, which shows that the unliganded enzyme no longer displays the hysteresis observed in hUGDH:UX4O (Figure 7A). Since hysteresis is a result of the slow transition from the E^Ω^ to the E state,^14−16^ the lack of a lag in unliganded hUGDH indicates the absence of a significant E^Ω^ population and is consistent with the cryo-EM data we have presented thus far. Preincubating unliganded hUGDH with UDP-Xyl or UX4O favors the E^Ω^ state and restores the lag in progress curves.

### UDP-Xylose and UDP-4-Keto-xylose Have Similar Affinities for hUGDH

The substrate saturation curves of hUGDH:UX4O display negative cooperativity with respect to NAD^+^ (Hill = 0.59 ± 0.04), consistent with previously published results (Figure 7B, Table 2).^13,15−17,19^ Fitting the data to Kurganov’s equation for negative cooperativity^43^ (eq 4) gives a *K*_0_ of 46.7 ± 9.9 μM and *K*_lim_ of 1179 ± 404 μM, where *K*_0_ approximates the *K*_M_ for the high-affinity site and *K*_lim_ represents the low affinity binding (Figure 7B inset, Table 2). Unliganded hUGDH also shows negative cooperativity with respect to NAD^+^(Hill = 0.77 ± 0.07), albeit to a lesser extent than hUGDH:UX4O (Figure 7C, Table 2). The Kurganov analysis suggests that apparent affinities for NAD^+^ have also increased, with a *K*_0_ of 24.9 ± 2.3 μM and *K*_lim_ of 143.7 ± 13 μM (Figure 7C, Table 2). Previously, we have shown that binding of NAD^+^ will induce the transition from E^Ω^ to the E state.^13,14^ Thus, it is likely that the small apparent increase in the *K*_M_ of hUGDH:UX4O is a function of the higher concentration of NAD^+^ needed to drive the UX4O-stabilized E^Ω^ complex to the E state. Similarly, the difference in Hill values between hUGDH and hUGDH:UX4O may indicate that the prebound UX4O is exaggerating an existing asymmetry in the NAD^+^ binding sites. In fact, the cryo-EM structure of hUGDH:UX4O clearly shows asymmetry in the ordering of the individual NB domains, which could contribute to the observed negative cooperativity (Figure 2A).

**Table 2:** Steady-State Parameters^a^,^b^,^c^

**enzyme**	**substrate**	***K***_**M**_**(μM)**	**Hill**	***k***_**cat**_ **(s**^**–1**^**)**^c^	***K***_**0**_^c^	***K***_**lim**_^c^	***k***_**cat**_^c^
hUGDH:UX4O	UDP-Glc	21 ± 0.6	1	1.2 ± 0.01			
	NAD^+^	1475 ± 426	0.59 ± 0.04	1.76 ± 0.14	46.7 ± 9.9	1179 ± 404	1.33 ± 0.17
hUGDH	UDP-Glc	10.5 ± 0.6	1	1.34 ± 0.01			
	NAD^+^	83.4 ± 10.4	0.77 ± 0.07	1.42 ± 0.04	24.9 ± 2.3	143.7 ± 13	1.39 ± 0.03

a Standard errors are reported.

b For calculating *k*_cat_, one complete catalytic turnover produces 2 NADH.

c Results from Kurganov analysis of a negatively cooperative enzyme.

The UDP-Glc substrate saturation curves for hUGDH:UX4O and hUGDH are both hyperbolic, with *K*_M_ values of 21 ± 0.6 and 10 ± 0.6 μM, respectively (Figure 7D,E, Table 2). The higher apparent *K*_M_ for hUGDH:UX4O is expected given the presence of the copurifying competitive inhibitor UX4O. We also measured the relative affinities of UX4O and UDP-Xyl for unliganded hUGDH using inhibition studies. The substrate saturation curves of unliganded hUGDH become increasingly sigmoidal with higher UX4O concentrations (Figure 7F). Previously, this sigmoidicity has been attributed to the substrate-induced conformational change from the low UDP-Glc affinity E^Ω^ complex to the higher affinity E state.^14^ Global analysis of the kinetic data yields a *K*_*i*_ of 0.75 ± 0.6 μM for UX4O (Figure 7F, Table 3). Similarly, UDP-Xyl inhibition of hUGDH also displays cooperativity, but with a lower *K*_*i*_ of 0.44 ± 0.02 μM. The similar turnover numbers and kinetic parameters of hUGDH and hUGDH:UX4O is strong evidence that the high pH sodium tetraborate buffer treatment used to remove the copurifying UX4O does not adversely affect the structure of the protein (Table 2).

**Table 3:** Global Analysis of Competitive Inhibition^c^

**enzyme**	UDP-Xyl (μM)	**UX4O (μM)**	**UDP-Glc*****K***_**M**_**(μM)**	***k***_**cat**_**(s**^**–1**^**)**^a^	***K***_***i***_**(μM)**	**Hill**
**hUGDH**	0		10.75 ± 0.45^b^	1.36 ± 0.01^b^	0.44 ± 0.02	0.93 ± 0.04
	1					1.34 ± 0.04
	2					1.56 ± 0.06
**hUGDH**		0	10.36 ± 0.64^b^	1.33 ± 0.02^b^	0.75 ± 0.06	0.98 ± 0.06
		1				1.03 ± 0.05
		2				1.14 ± 0.06

a For calculating *k*_cat_, one complete catalytic turnover produces 2 NADH.

b For global analysis, this parameter was refined as a shared value for all UDP-Xyl concentrations.

c Standard errors are reported.

## Discussion

Our previous crystal structures suggested that unliganded hUGDH favored the E state,^12,14,17^ but this interpretation was not supported by transient-state analysis. Specifically, the hysteresis in hUGDH progress curves arises from the slow transition of the inactive E^Ω^ to the active E state, and our earlier estimates based on modeling the transition predicted ∼90% of the unliganded enzyme would occupy the E^Ω^ state at physiological pH (which is near the pH of the crystal structures).^14^ Here we have used cryo-EM studies to resolve the discrepancy between the earlier crystal structures and the kinetic data. The cryo-EM reconstruction of recombinant hUGDH revealed the major species was the E^Ω^ state, which is consistent with the earlier transient-state model for the hysteresis in progress curves (Figure 2A). However, both the reconstruction and subsequent biochemical analysis showed that hUGDH expressed in *E. coli* copurifies with a tightly bound UX4O (Figures 3 and 4). UX4O is structurally similar to UDP-Xyl and it triggers the same allosteric transition that forms the E^Ω^ state (Figures 3). But if hUGDH purified from *E. coli* is saturated with UX4O and adopts the E^Ω^, why did the earlier structural studies yield unliganded E state UGDH?^14,17,19^ One reason for the difference between the cryo-EM and crystallographic studies could be variability in the amount of UX4O contamination in different protein preparations. However, we have not seen any significant variation in the amount of UX4O contamination in the recent protein preparations that we have assayed (Figure 3D). Even the protein used in earlier crystal structure studies displayed lags in progress curves, which we now know is evidence of the E^Ω^ state stabilized by UX4O or UDP-Xyl binding (Figure 7A). It is more likely that the earlier, unliganded E state crystal structures were a result of *the tyranny of the lattice*(47) Briefly, if the crystal lattice selected for the E state conformation, then it would likely favor the dissociation of the hUGDH:UX4O complex, especially in the absence of a saturating concentration of UX4O added to the crystallization drop. We have previously shown^15,16^ that the E state has a lower affinity for UDP-Xyl, which is presumably also true for UX4O based on the conserved binding interactions and similar *K*_*i*_ values (Figure 5A and Table 3). The open NB domains seen in the unliganded E state crystal structure would also favor UX4O dissociation. This conclusion is supported by the improvement of UX4O density when comparing the flexible to the ordered NB domains of the Omega arms and bridging dimers, respectively (Figure 3C), and in the results of the focused refinement showing that closing the NB_exposed_ domain results in stronger UX4O density (not shown). Interestingly, in one of our previous crystal structures of unliganded hUGDH, (PDB ID 5TJH) we did report a weakly ordered nucleotide sugar bound in one of the subunits that had copurified with enzyme.^15^ At the time, we tentatively identified the ligand as UDP-Glc, but considering our new results, it is possible that the contaminant was actually UX4O. We have also recently identified UX4O copurifying with *E. coli* expressed UGDH from both *Caenorhabditis elegans* and *Petromyzon marinus*, suggesting that UX4O contamination is a common feature in recombinantly expressed allosteric UGDHs (data not shown). In fact, Dickinson previously purified UGDH from beef liver and reported the presence of a copurifying nucleotide and a lag in progress curves.^48^ While he was not able to unambiguously identify the nucleotide, he did note that it behaved most like UDP-Xyl in HPLC analysis. Based on our work here, it would not be surprising to find UDP-Xyl copurifying in liver-derived UGDH given that it is the native allosteric inhibitor of mammalian UGDH. Dickinson also showed that the hysteresis in bovine liver UGDH vanished if the enzyme was preincubated at pH 9, which is consistent with our observation that UGDH releases UX4O at high pH (Figure 4A). Thus, when purifying allosteric UGDH, it is important to be aware that UX4O or UDP-Xyl are likely contaminants, and the column wash steps should incorporate a high pH wash to remove the nucleotide sugar.

Our work and that of others^34,35,45,46^ show that UX4O readily hydrates to form a geminal-diol (UGDX) in aqueous solution (Figure 4C,D). Thus, it was surprising to observe UX4O and not UGDX in the active site of the hUGDH cryo-EM structure (Figure 5). While UX4O shares most of the same binding interactions as UDP-Xyl, there is an important difference involving the corresponding C4 positions; in UX4O, the C4 ketone oxygen introduces an unfavorable electrostatic repulsion with the backbone carbonyl oxygen of E161, but in the case of xylose, the C4 hydroxyl forms a favorable hydrogen bond with same residue (Figure 5). The discovery of such a significant difference in binding interactions between the two inhibitors was unexpected, because inhibition studies showed that UX4O and UDP-Xyl have similar affinities for hUGDH (*K*_*i*_ of 0.75 and 0.44 μM, respectively) (Table 3). This can be explained if the dehydration of UGDX upon binding in the active site of hUGDH is sufficiently favorable that it could offset the unfavorable electrostatic repulsion between the ketone and backbone carbonyl oxygens. In support of this hypothesis, it is known that the majority of gem diols are unstable, and only form in aqueous solutions.^49^ In fact, we have shown that UGDX rapidly dehydrates in the vacuum of a mass spectrometer to produce UX4O (Figure 4E).

Our results show that UX4O can function as an alternative allosteric inhibitor of hUGDH, but is it physiologically relevant? In an earlier phylogenetic study,^19^ we identified putative allosteric UGDHs in bacteria, suggesting that this feedback inhibition mechanism is ancient. While there are relatively few reports of xylose in bacterial glycans, it is known that many diverse bacterial species contain UDP-GlcA decarboxylases like ArnA and can produce UX4O.^50^ Thus, it is possible that UX4O serves as a feedback inhibitor in bacterial species that possess an allosteric UGDH but do not make UDP-Xylose. Unfortunately, it is difficult to use sequence-based methods alone to distinguish UX4O producing UDP-glucuronic acid decarboxylases from those that make UDP-Xylose.^18,50^ Future biochemical studies will be needed to confirm our hypothesis, but if true, it may be that UX4O is an ancient allosteric inhibitor of UGDH.

## Conclusions

UX4O is a metabolite of *E. coli* that is structurally similar to UDP-xylose, the native allosteric inhibitor of hUGDH. This study shows that UX4O is a common contaminant of recombinantly expressed hUGDH preparations, and can remain tightly bound throughout IMAC purification, extensive dialysis and size exclusion chromatography. Leveraging the strong pH dependency of UDP-xylose inhibition, we have developed a new purification protocol that produces unliganded hUGDH and pure UX4O. Removing the contaminating UX4O solves a long-standing discrepancy over which conformational state is favored by the unliganded enzyme. Specifically, previous kinetic analysis suggested that unliganded hUGDH favors the E^Ω^ state, which contrasted with structural studies supporting the active E conformation as the dominant species. Here, we show that UX4O shifts the ensemble toward the E^Ω^ state, and that unliganded hUGDH strongly favors the E state. Using the cryo-EM structures of pure hUGDH and UX4O-bound hUGDH, we have also analyzed the allosteric response in the absence of the constraints of a crystal lattice. The E^Ω^ reconstruction reveals a high degree of flexibility for the N*B*_exposed_ domains in the omega arm dimers that was not observed in the existing crystal structures. This domain flexibility combined with the observed substoichiometric binding of the UX4O in purified hUGDH suggests that the affinity for the inhibitor in the two NB_exposed_ domains is likely lower than that of the other subunits.

## Supporting Information Available

Figures S1–S4: Cryo-EM refinement workflow; Figure S5: NMR analysis of UDP-4-Geminal-Diol-Xylose (UGDX) (PDF)
