# Supplementary material for: Cryo-EM Structure of Recombinantly Expressed hUGDH Unveils a Hidden, Alternative Allosteric Inhibitor
Source: Biochemistry. 2024 Dec 16;64(1):92–104. doi: 10.1021/acs.biochem.4c00555 (PMC11713868; doi:10.1021/acs.biochem.4c00555)
Supplement: Supplementary file 1 — bi4c00555_si_001.pdf [file bi4c00555_si_001.pdf]

Supporting Information

The Cryo-EM structure of recombinantly expressed hUGDH unveils a hidden, alternative allosteric inhibitor

John H. O'Brien[a], Renuka Kadirvelraj[a], Po-Sen Tseng[b], Nolan Ross-Kemppinen[a], David Crich[b], Richard M. Walsh, Jr.[c]* and Zachary A. Wood[a]*

[a] *Department of Biochemistry & Molecular Biology, University of Georgia, Athens GA 30602, USA*

[b] *Department of Pharmaceutical and Biomedical Sciences, Department of Chemistry, and Complex Carbohydrate Research Center, University of Georgia, Athens, GA 30602, USA*

[c] *Department of Biological Chemistry and Molecular Pharmacology, Blavatnik Institute, Harvard Medical School, Boston, MA 02115, USA*

* For editorial correspondence: Zachary A. Wood, phone: 706-583-0304, FAX: 706-542-1738, email: zaw@uga.edu and Richard M. Walsh, Jr. phone: 617 432-9042, email: richard_walsh@hms.harvard.edu

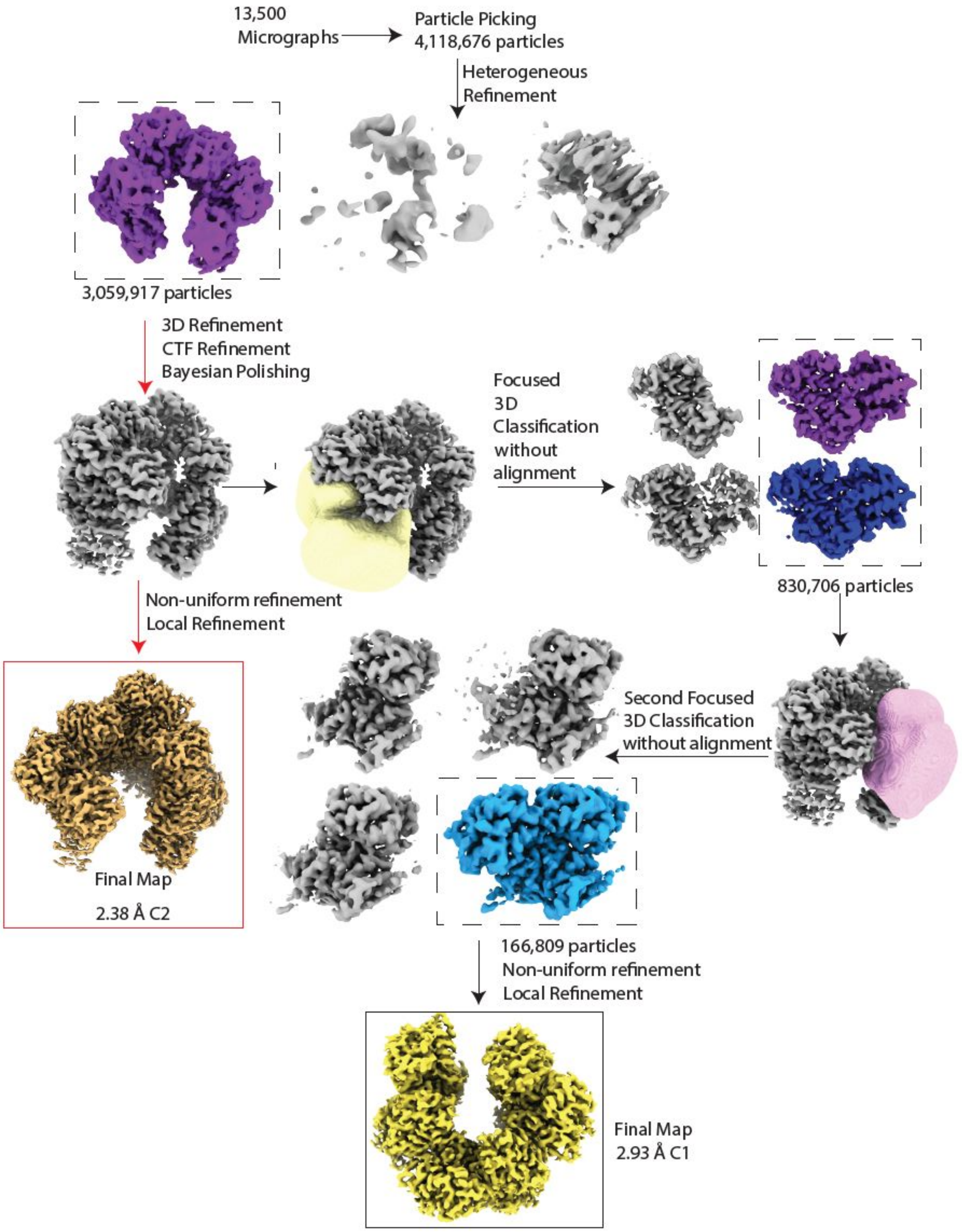

Supplementary Figure 1: Cryo-EM processing scheme for the classification and refinement of nucleotide bound hUGDH sample. Approximately 95% of the data led to a 2.4 Å map for the hUGDH:UX4O model with disordered NAD^+ domains for the two arm dimers of the E^{Ω} complex (boxed in red). Using focused classification, a smaller subset of the data (~5%) with better order for the NAD^+ domains of the two arm dimers was identified (boxed in black).

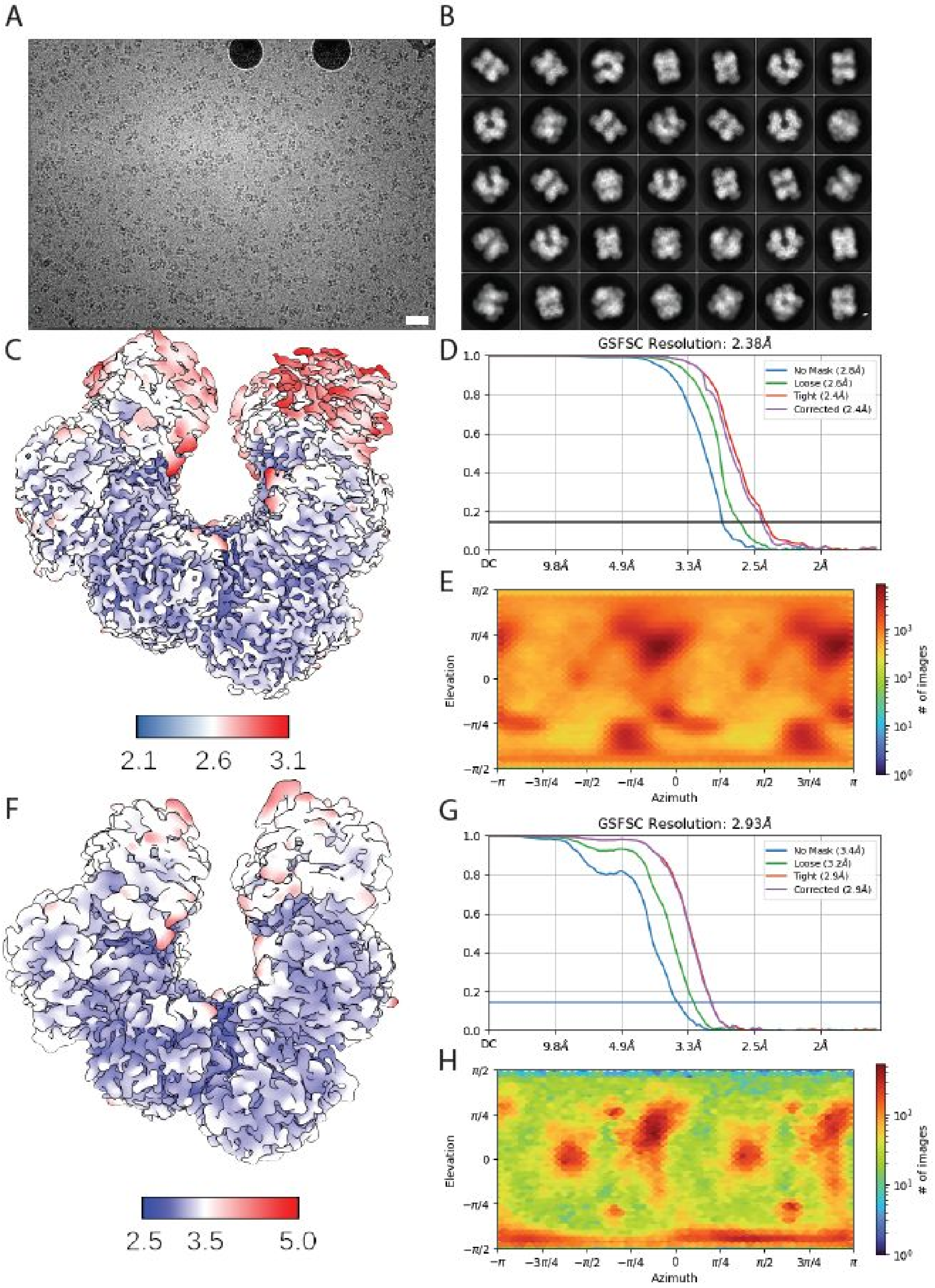
A
B
C
D
GSFSC Resolution: 2.38Å
No Mask (2.6Å)
Loose (2.6Å)
Tight (2.4Å)
Corrected (2.4Å)
DC
9.8Å
4.9Å
3.3Å
2.5Å
2Å
2.1
2.6
3.1
E
Elevation
Azimuth
# of images
F
G
GSFSC Resolution: 2.93Å
No Mask (3.4Å)
Loose (3.2Å)
Tight (2.9Å)
Corrected (2.9Å)
2.5
3.5
5.0
H

Supplementary Figure 2: Cryo-EM data analysis of nucleotide bound hUGDH. A) Representative micrograph of hUGDH:UX4O embedded in vitreous ice (scale bar = 500 Å), low pass filtered for clarity. Scale bar (white) is 500 Å. **B)** Selected 2D class averages of hUGDH:UX4O. **C)** Higher resolution reconstruction of hUGDH:UX4O filtered and colored by local resolution. Panels D and E refer to the higher resolution reconstruction depicted in Panel C. **D)** Gold-standard Fourier shell correlation (FSC) curves from cryoSPARC. **E)** Viewing direction distribution plot. **F)** Lower resolution, but more complete reconstruction of hUGDH:UX4O filtered and colored by local resolution. Panels G and H refer to the lower resolution reconstruction depicted in Panel F. **G)** Gold-standard Fourier shell correlation (FSC) curves from cryoSPARC. **H)** Viewing direction distribution plot.

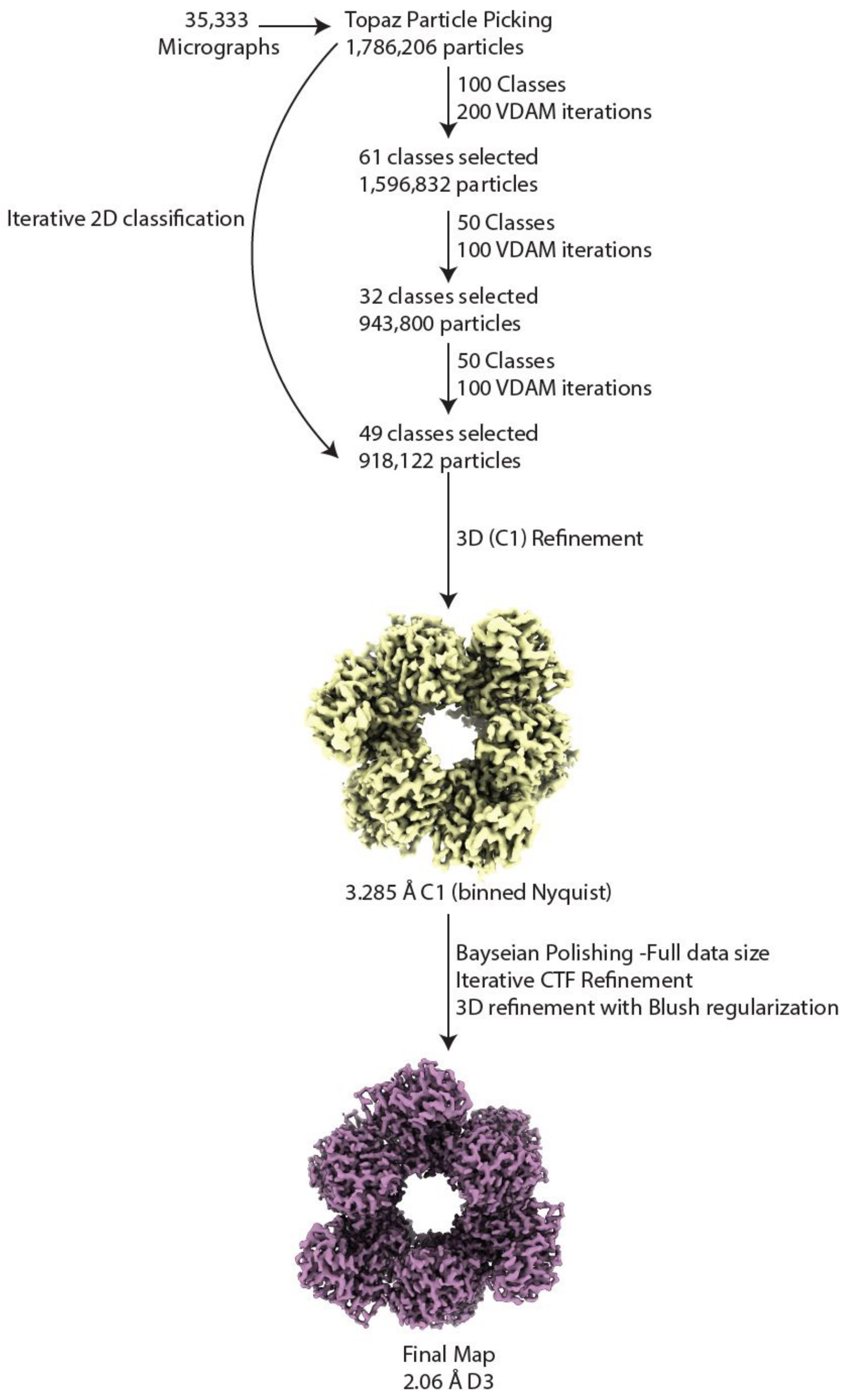

Supplementary

Figure 3: Cryo-EM processing procedure for unliganded hUGDH sample. Processing scheme for classification and refinement of unliganded hUGDH.

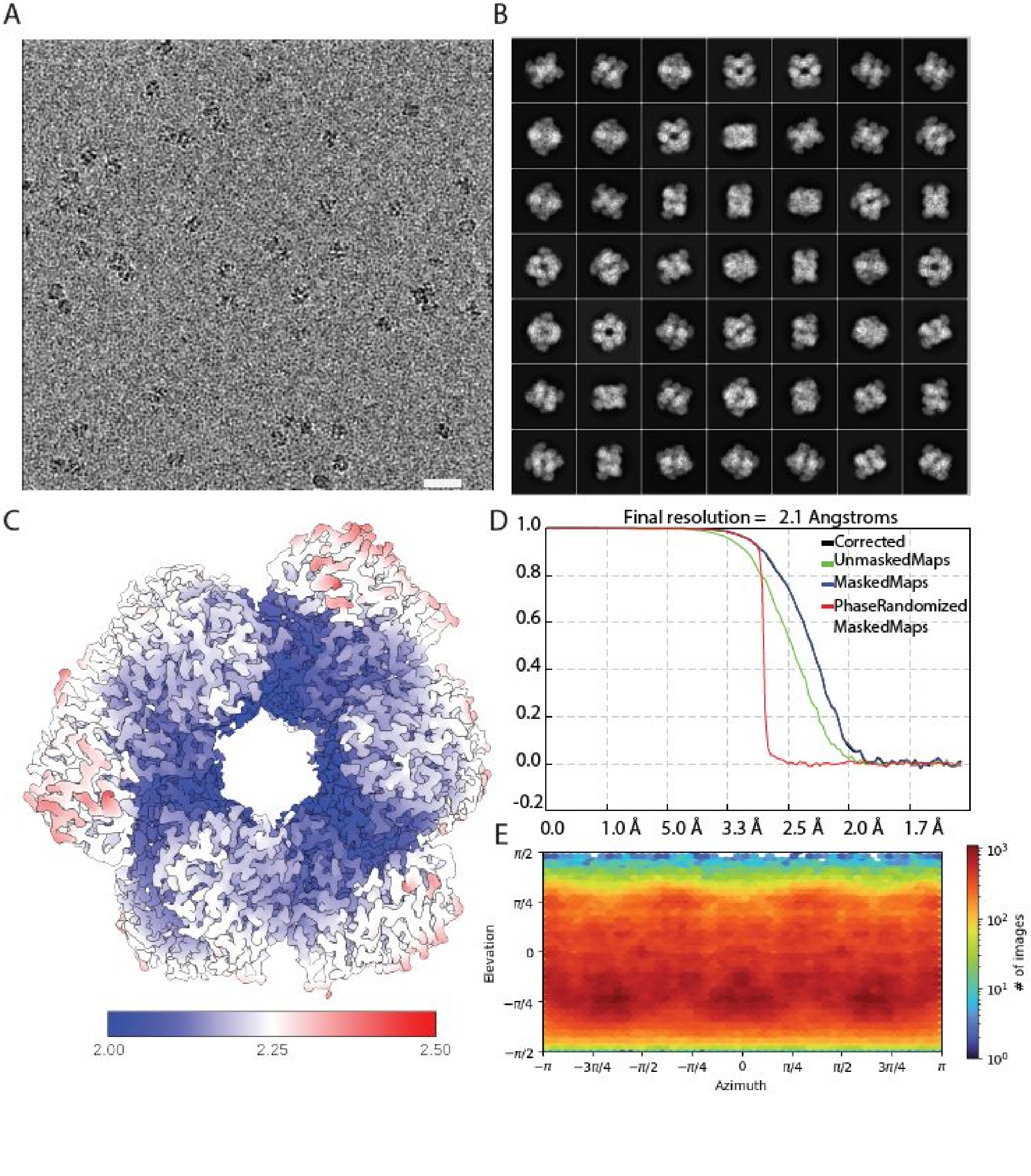

Supplementary Figure 4: Cryo-EM data analysis of unliganded hUGDH sample. A) Representative micrograph of hUGDH embedded in vitreous ice (scale bar = 500 Å), low pass filtered for clarity. Scale bar (white) is 5000 Å. **B)** Selected 2D class averages of hUGDH. **C)** Reconstruction of hUGDH filtered and colored by local resolution. **D)** Gold-standard Fourier shell correlation (FSC) curves from Relion. **E)** Viewing direction distribution plot. Data were subjected to Non-Uniform refinement in cryoSPARC to generate Viewing direction distribution plot for comparison to the nucleotide bound dataset.

Supplementary Figure 5:

Characterization Data of UDP-4-Geminal-Diol-Xylose (UGDX)

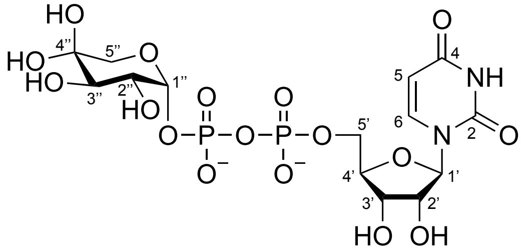

^{1}H NMR (600 MHz, H_2O+D_2O): δ 7.98 (d, *J* = 8.1 Hz, 1H, Ura H-6), 6.01 – 5.98 (m, 2H, Rib H-1' and Ura H-5), 5.60 (dd, *J* = 7.1, 3.5 Hz, 1H, GDX H-1''), 4.40 – 4.38 (m, 2H, Rib H-2' and Rib H-3'), 4.32 – 4.29 (m, 1H, Rib H-4'), 4.26 (ddd, *J* = 11.8, 4.5, 2.5 Hz, 1H, Rib H-5a'), 4.21 (ddd, *J* = 11.8, 5.6, 2.8 Hz, 1H, Rib H-5b'), 3.95 (d, *J* = 12.2 Hz, 1H, GDX H-5a''), 3.83 (d, *J* = 9.8 Hz, 1H, GDX H-3''), 3.67 (ddd, *J* = 9.8, 3.5, 3.1 Hz, 1H, GDX H-2''), 3.57 (d, *J* = 12.2 Hz, 1H, GDX H-5b'').

^{13}C NMR (151 MHz, H_2O+D_2O): δ 166.2 (Ura C-4), 151.8 (Ura C-2), 141.6 (Ura C-6), 102.7 (Ura C-5), 95.9 (d, *J* = 6.6 Hz, GDX C-1''), 92.6 (GDX C-4''), 88.3 (Rib C-1'), 83.2 (d, *J* = 9.2 Hz, Rib C-4'), 73.8 (Rib C-2'), 72.6 (GDX C-3''), 70.5 (d, *J* = 8.8 Hz, GDX C-2''), 69.6 (Rib C-3'), 65.4 (GDX C-5''), 64.9 (d, *J* = 5.6 Hz, Rib C-5').

HRMS–ESI (*m/z*): $[M–H]^-$ calcd for $C_{14}H_{19}O_{16}N_2P_2$, 533.0215; found, 533.0223.

^{1}H NMR (600 MHz, H_2O+D_2O) Spectrum of UDP-4-Geminal-Diol-Xylose (UGDX)

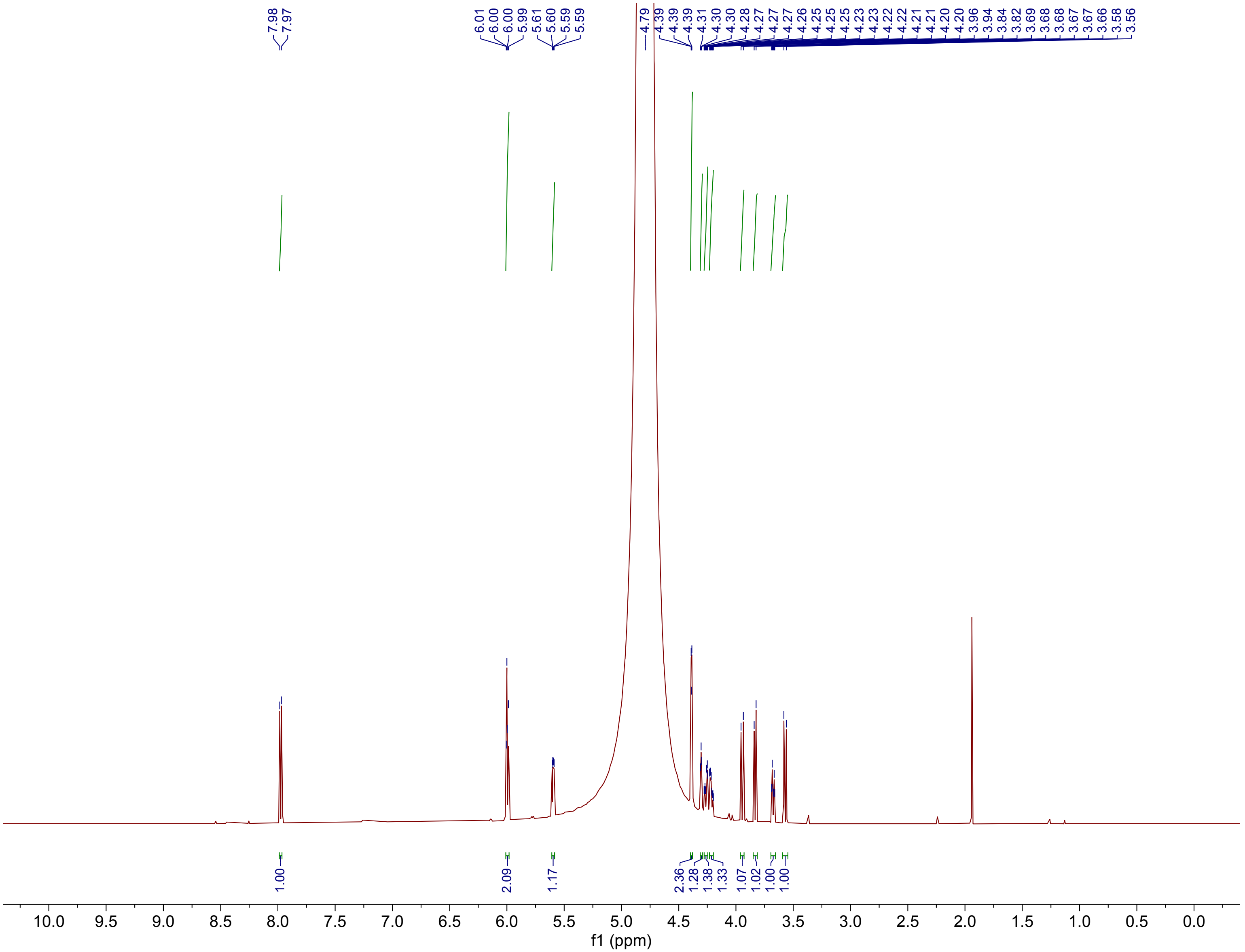

^{13}C NMR (151 MHz, H_2O+D_2O) Spectrum of UDP-4-Geminal-Diol-Xylose (UGDX)

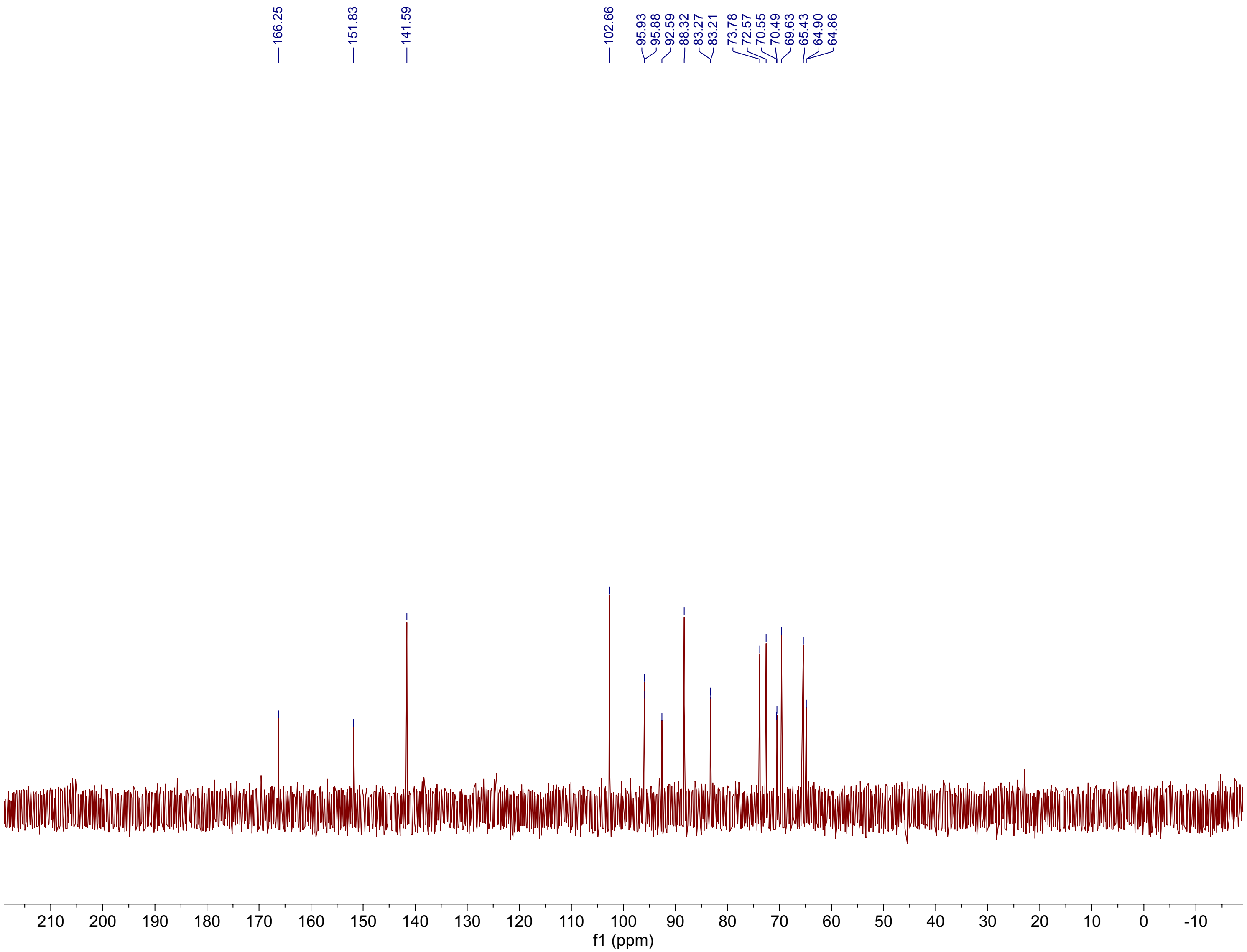

COSY NMR (600 MHz, H_2O+D_2O) Spectrum of UDP-4-Geminal-Diol-Xylose (UGDX)

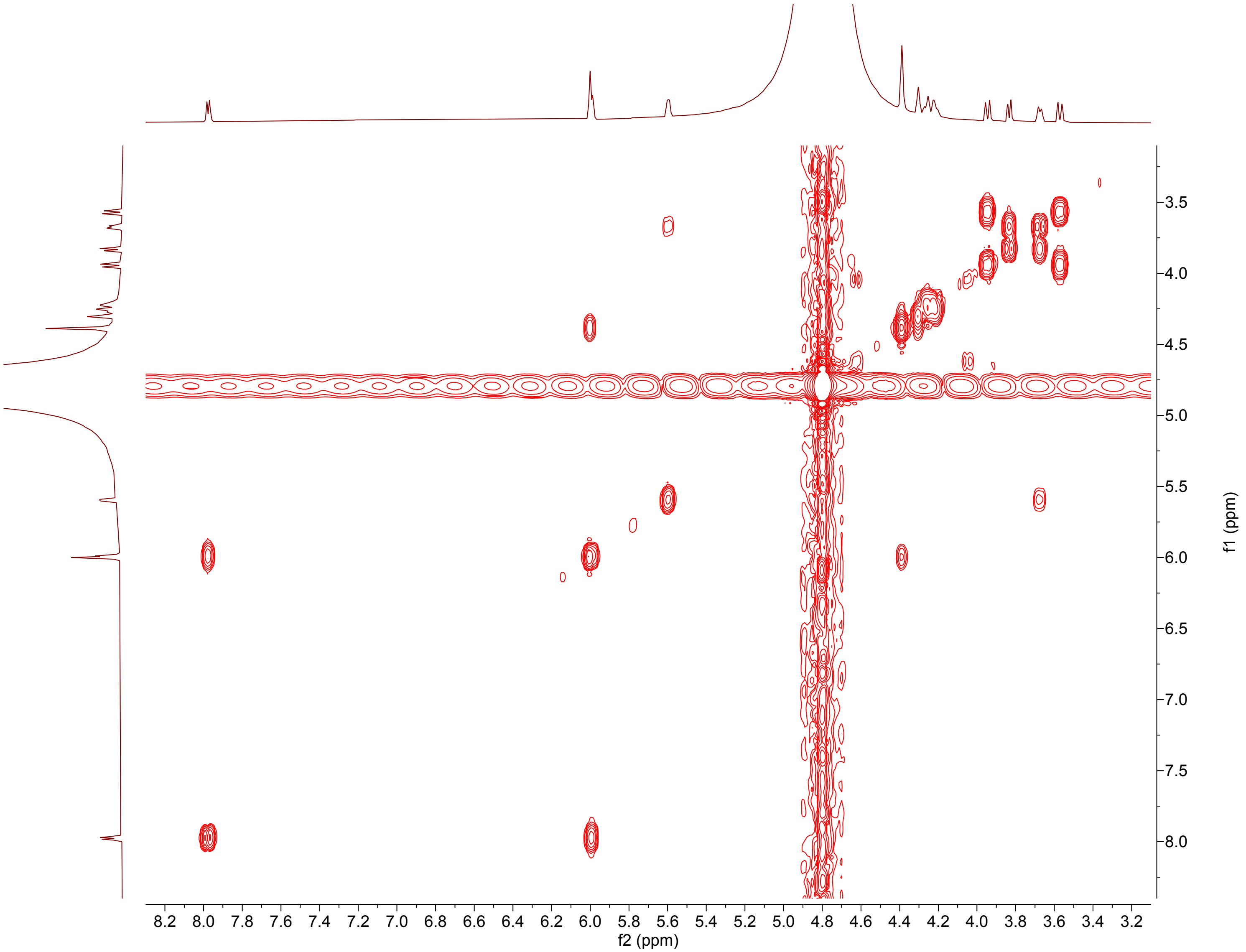

HSQC NMR (600 MHz, H_2O+D_2O) Spectrum of UDP-4-Geminal-Diol-Xylose (UGDX)

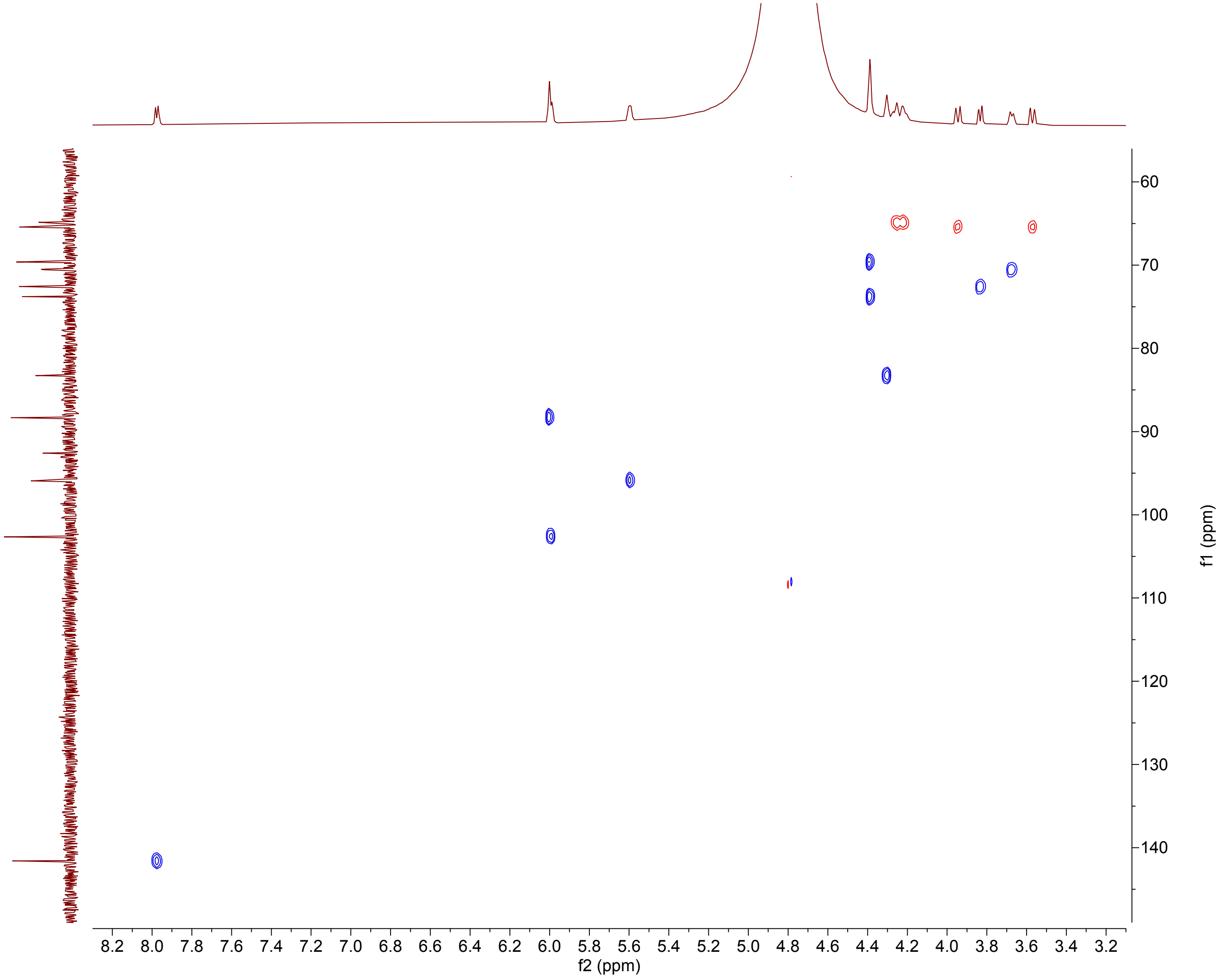

HMBC NMR (600 MHz, H_2O+D_2O) Spectrum of UDP-4-Geminal-Diol-Xylose (UGDX)

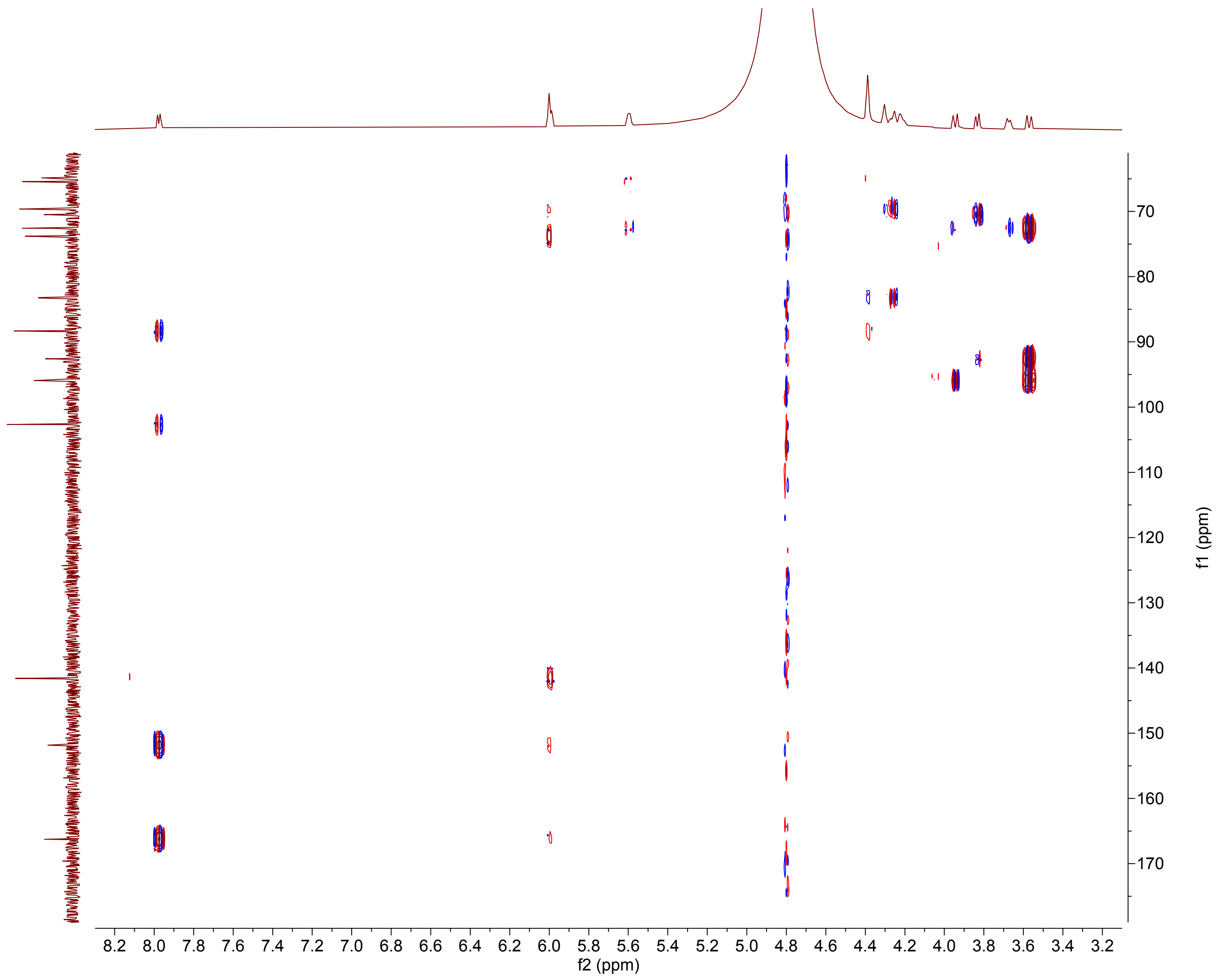

Associated Content

PDB and EMDB codes:

- hUGDH:UX4O - PDB ID: 9DH0, EMDB code: EMD-46854
- hUGDH - PDB ID: 9DGZ, EMDB code: EMD-46853
